# Diverse midbrain dopaminergic neuron subtypes and implications for complex clinical symptoms of Parkinson’s disease

**DOI:** 10.20517/and.2021.07

**Published:** 2021-07-15

**Authors:** Kathleen Carmichael, Breanna Sullivan, Elena Lopez, Lixin Sun, Huaibin Cai

**Affiliations:** 1 Transgenic Section, Laboratory of Neurogenetics, National Institute on Aging, National Institutes of Health, Bethesda, MD 20892, USA.; 2 The Graduate Partnership Program of NIH and Brown University, National Institutes of Health, Bethesda, MD 20892, USA.

**Keywords:** Parkinson’s disease, ageing, dopaminergic neurons, dopamine, SNc, VTA, ALDH1A1, RNA sequencing, subpopulation

## Abstract

Parkinson’s disease (PD), the most common degenerative movement disorder, is clinically manifested with various motor and non-motor symptoms. Degeneration of midbrain *substantia nigra pas compacta* (SNc) dopaminergic neurons (DANs) is generally attributed to the motor syndrome. The underlying neuronal mechanisms of non-motor syndrome are largely unexplored. Besides SNc, midbrain ventral tegmental area (VTA) DANs also produce and release dopamine and modulate movement, reward, motivation, and memory. Degeneration of VTA DANs also occurs in postmortem brains of PD patients, implying an involvement of VTA DANs in PD-associated non-motor symptoms. However, it remains to be established that there is a distinct segregation of different SNc and VTA DAN subtypes in regulating different motor and non-motor functions, and that different DAN subpopulations are differentially affected by normal ageing or PD. Traditionally, the distinction among different DAN subtypes was mainly based on the location of cell bodies and axon terminals. With the recent advance of single cell RNA sequencing technology, DANs can be readily classified based on unique gene expression profiles. A combination of specific anatomic and molecular markers shows great promise to facilitate the identification of DAN subpopulations corresponding to different behavior modules under normal and disease conditions. In this review, we first summarize the recent progress in characterizing genetically, anatomically, and functionally diverse midbrain DAN subtypes. Then, we provide perspectives on how the preclinical research on the connectivity and functionality of DAN subpopulations improves our current understanding of cell-type and circuit specific mechanisms of the disease, which could be critically informative for designing new mechanistic treatments.

## INTRODUCTION

Parkinson’s disease (PD) is the second most common degenerative neurological disorder affecting millions of elderly individuals worldwide. PD patients display canonical motor symptoms, including resting tremor, slowed movement, impaired posture and balance, and rigid muscles^[1]^. The motor syndrome is generally regarded as the result of extensive loss of nigrostriatal dopaminergic neurons (DANs) in the *substantia nigra pars compacta* (SNc) of the midbrain^[2,3]^. Dopamine replacement medications and deep brain stimulation surgery can improve some of the patient’s motor conditions. However, no cure is available. Moreover, long-term medication can cause severe side effects, such as dyskinesia and impulsive control disorders^[4]^. New mechanistic insights and therapeutic agents are still needed to improve patients’ treatment and life quality.

In addition to motor symptoms, PD patients often suffer from depression, dementia, and other neuropsychiatric symptoms^[5]^. For example, PD patients often develop cognitive dysfunctions, which leads to Parkinson’s disease dementia (PDD)^[6,7]^. Approximately 75% of PD patients develop dementia within 10 years of diagnosis, and the prevalence of PDD is 0.3%-0.5% in the general population older than 65 years^[6]^. The exact pathogenic mechanisms of PDD and other PD-related non-motor symptoms are largely unknown. The cognitive dysfunctions are not improved by levodopa, the most effective drug to treat the motor symptoms in PD^[6]^. Therefore, an important step in intervening a complex neurological disorder such as PD is to fully elucidate the functional roles of different neural circuits responsible for specific behavioral phenotypes.

It has been generally accepted that ageing, environmental toxins, and genetic mutations contribute to the etiopathogenesis of PD. Ageing is the most significant risk factor in the development of PD and other neurodegenerative diseases. Genomic instability, epigenetic alterations, loss of proteostasis, mitochondrial dysfunction, and altered intracellular communication are among the key ageing hallmarks^[8]^. Various environmental toxins and genetic risk factors have been linked to PD, which affect largely overlapping molecular and cellular pathways as those implicated in ageing^[9-11]^. However, the molecular genetic studies often fall short in pinpointing any specific cell-types or neural circuits critical for the alterations and impairments of motor and non-motor behaviors. To better understand how different brain cells and neural circuits control diverse behaviors will therefore provide the structural framework to better appreciate the impacts of ageing and environmental and genetic factors on the cause and progression of the disease-related behavioral abnormalities.

The midbrain DANs are composed of diverse neuron subpopulations based on the location of cell bodies, projection patterns, morphology, gene expression profiles, electrophysiological properties, physiological functions, and vulnerabilities to diseases^[12-17]^. Since a preferential degeneration of midbrain DANs represents the most significant neuropathological feature of PD, in this review, we focus our discussion mainly on the diversity of midbrain DANs in their distinct genetic makeups, connectivity, and functionality. Because animal research has been instrumental in understanding the pathophysiological mechanisms and developing the treatments of PD, such as the current dopamine replacement therapy and deep brain stimulation surgery^[18]^, we mainly summarize the research findings from preclinical animal models.

## MOLECULAR GENETIC DIVERSITY OF MIDBRAIN DOPAMINERGIC NEURONS

Midbrain DANs, residing in the ventral region of the midbrain, are grouped together based on their ability to synthesize and release dopamine, a key neuromodulator involved in motor control and learning, motivation, cognition, and reward^[19,20]^. Dysfunction of midbrain DAN-mediated dopamine transmission has been associated with PD, schizophrenia, addiction, and other neurological and psychological disorders^[5,21,22]^. Traditionally, midbrain DANs can be divided into three main subgroups, retrorubral field (RRF, A8), SNc (A9), and ventral tegmental area (VTA, A10), in humans and rodents^[23,24]^. The midbrain DANs also differ in their axon projections to different brain regions and physiological functions. In addition, the SNc DANs are relatively more vulnerable to neuronal toxins in rodent PD models and preferentially degenerated in PD patients^[25,26]^. However, molecular genetic makers are needed to better characterize different DAN subtypes, as well as their distinctive connectivity and functionality.

The midbrain DANs selectively express genes critical for the dopamine synthesis, transport, and degradation, such as tyrosine hydrolase (TH), vesicular monoamine transporter 2 (VMAT2), dopamine transporter (DAT), and aldehyde dehydrogenase 1a1 (ALDH1A1)^[27]^. The midbrain DANs also express transcription factors critical for the DAN differentiation and survival, including nuclear receptor related 1 protein (NURR1), pituitary homeobox 3 (PITX3), and forkhead box protein A1/2 (FOXA1/2)^[28,29]^. The molecular genetic difference between SNc and VTA DANs was initially studied by immunostaining and *in situ* hybridization using preselected genetic makers. Those markers included G-protein activated inwardly rectifying potassium channel 2 (GIRK2/KCNJ6), which is more abundant in SNc DANs^[30,31]^, and calbindin (D-28K, CALB1), which is more enriched in VTA DANs^[32-34]^. However, those genetic markers are not exclusively expressed by the SNc or VTA DANs. Additional genetic markers are required to specifically investigate the functions of different SNc and VTA DAN subtypes. The whole-genome gene expression studies using microarray and RNA sequencing (RNAseq) technology, especially the latest single cell RNAseq (scRNAseq) techniques, in combination with Laser Capture Microdissection (LCM) and Fluorescence-Activated Cell Sorting (FACS) procedures, provide the means to systematically identify distinct genetic identifiers for diverse DAN subpopulations^[35]^.

### Laser capture microdissection, fluorescence-activated cell sorting, and microarray studies of differential gene expression between SNc and VTA DANs

The TH-positive DANs in SNc and VTA were visually marked with a so-called rapid immunostaining procedure, and then collected separately by LCM. RNAs were extracted from the LCM-isolated SNc and VTA samples and subjected to microarray analyses. In both rat^[36]^ and mouse^[37]^ studies, numerous differentially expressed genes were found between SNc and VTA DANs. Some of those differentially expressed genes were reported by both studies. For example, the expression of Igf1, Gad1, Drd2, and Sncg is higher in the SNc, while the expression of Otx2, Tacr3, and Lpl is higher in the VTA. However, very few genetic markers can be used to distinguish between the SNc and VTA DANs, indicating that the molecularly defined DANs may not always be confined within the anatomical boundaries. In our studies, while we found that the majority of ALDH1A1-positive DANs are distributed in the ventral tier of SNc, a minority population is scattered in a broad region in the VTA^[38,39]^. A combination of two or more genetic markers would be required to identify a distinct subtype of DANs in SNc or VTA. On the other hand, since there were non-dopaminergic cells within the LCM samples, additional immunostaining and *in situ* hybridization experiments are needed to verify the expression of any gene of interest in the DANs. Furthermore, because of difficulty in physically isolating small DAN clusters by LCM, the distinct gene expression profile of DAN subtypes can only be elucidated by the later FACS and scRNAseq techniques.

### Multiple subtypes of midbrain DAN identified by single-cell RNA-sequencing

**Figure 1.**
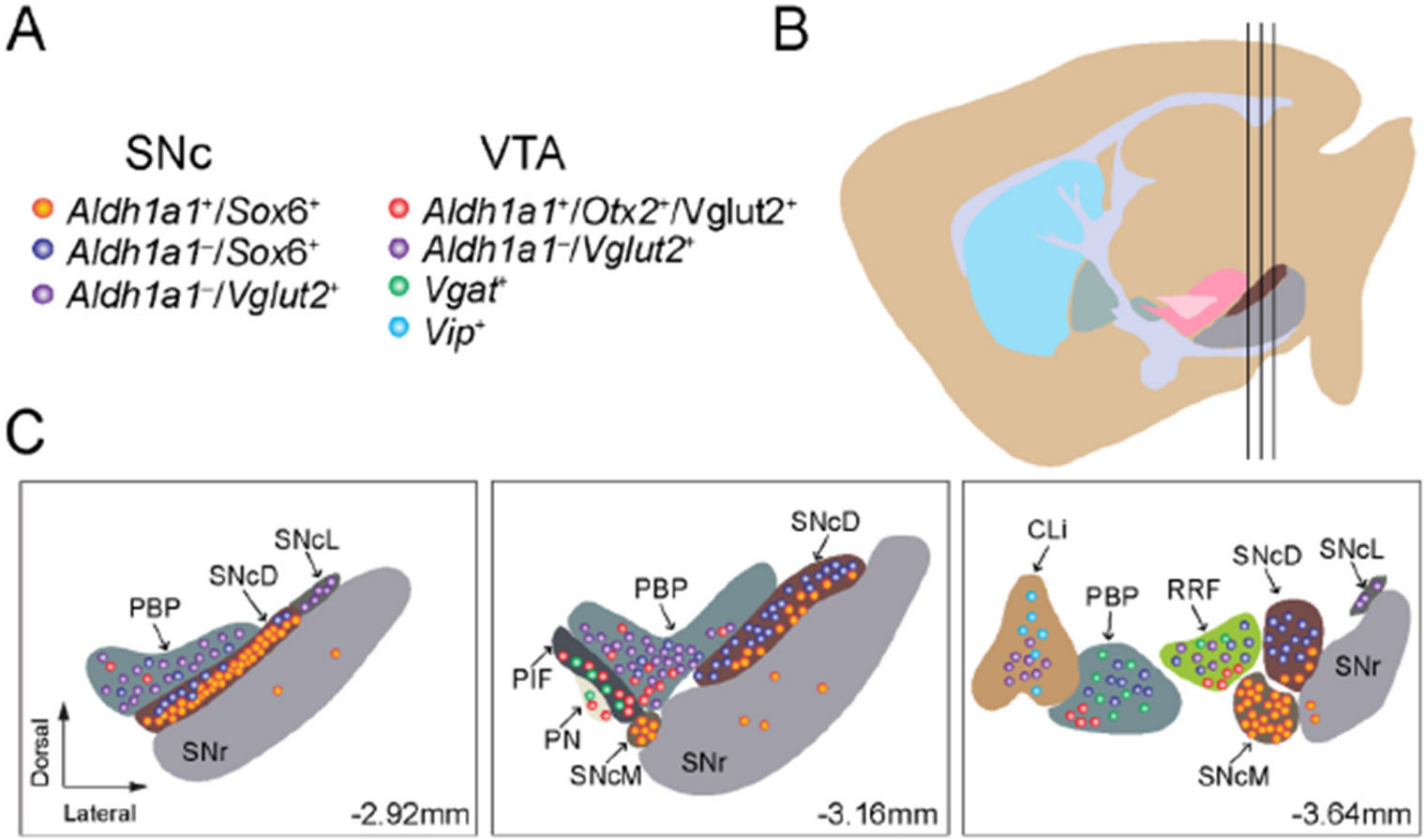
We outline the regional distribution of seven molecularly defined DAN subtypes in the SNc and VTA: (A) a list of molecularly defined DAN subtypes in the SNc and VTA; (B) a sagittal view of adult mouse brain where the three vertical black lines mark the positions of three cross-sections depicted in (C); and (C) regional distribution of molecularly defined DAN subtypes in the midbrain at Bregma-2.92, −3.16, and −3.64 mm. SNcD: SNc dorsal; SNcM: SNc medial; SNcL: SNc lateral; PBP: parabrachial pigmented nucleus; Cli: caudal linear nucleus of the raphe; PN: paranigral nucleus; PIF: parainterfascicular nucleus; SNc: *substantia nigra pars compacta*; VTA: ventral tegmental area.

By employing the newly available scRNAseq technology, recently multiple studies have been performed to reveal the diverse gene expression profiles of midbrain DANs at single cell level^[35]^. These high-resolution gene expression studies demonstrate more complex gene expression patterns in individual midbrain DANs, identify more DAN subtypes with additional genetic markers, and improve our understanding of the genetic diversity of midbrain DANs^[14,40-43]^. Based on distinct gene expression patterns, midbrain DANs may constitute about 10 or even more subtypes. However, different numbers of DAN subtypes were reported from different studies, which often used different subtype names and proposed different genetic markers for clustering the subtypes. These differences reflect different technical approaches and classification criteria in their studies, while presenting a challenge to other researchers to consistently define the major subtypes of midbrain DANs^[44]^. We highlighted seven major midbrain DAN subtypes and listed their unique classification criteria based on the published mouse scRNAseq studies [Figure 1]. The heterogenous gene expression profiles of midbrain DANs revealed by scRNAseq and verified by the follow-up RNA scope *in situ* hybridization demonstrate the distribution of distinct subgroups of DANs in the developing and adult mouse midbrains. For example, the ALDH1A1-positive SNc DANs, which are mainly located in the ventral tier of SNc, express high levels of Sox6, Th, Dat, Aldh1a7, Lmo3, Anxa1, and Sncg, but not Otx2 and CALB1^[40,43]^. By contrast, the ALDH1A1-positive VTA DANs, which are found to be intermingled with other cell types in the VTA, express high levels of CALB1 and Vglut2, but not Sox6^[14,17,40,43-46]^. Therefore, based on the co-expression of Sox6 or CALB1, ALDH1A1-positive DANs can be assigned to SNc or VTA subregions in the midbrain [Figure 1]. Further in-depth analyses of distinct gene expression in ALDH1A1-positive SNc or VTA DANs may reveal additional molecularly and anatomically defined subtypes responsible for distinct physiological functions. To reliably interpret scRNAseq data, future studies need to improve the sensitivity in detecting low-level gene expression, as well as increase the numbers of cells collected in each experiment.

## DIVERSE SNC DOPAMINERGIC NEURON SUBPOPULATIONS

The midbrain DAN system is the largest and most complex in primates compared to rodent species, with up to 600,000 TH-positive cells in humans compared to ~25,000 TH-positive cells in mice^[47]^. In rodents, about half of the TH-positive cells across A8, A9, and A10 are found within the substantia nigra (SN)^[47]^. In monkeys and humans, there is both a large increase in the number of TH-positive cells and an even larger percentage (> 70%) of the number of TH-positive cells that are located in the SN^[47]^. Beyond simply noting the number and percentage of SNc dopamine neurons across species, studies have also gone on to document the anatomic, molecular, and functional diversity of SNc DANs.

### Anatomic and molecular diversity of SNc dopaminergic neuron subtypes

The DANs in the midbrain of rodents and primates can also be classified into a dorsal tier and a ventral tier^[48-50]^. The SN itself can be further subdivided into the SNc and *substantia nigra pars reticulata* (SNr). DANs are very densely packed within the SNc, while neurons are more sparse and diffuse within the SNr^[51]^. The DANs in the SNc are well known for their projections to the dorsal striatum (which consists of the caudate nucleus and the putamen) in what is known as the nigrostriatal dopamine pathway^[52]^. It has been well established that the striatum can be divided into two neurochemically distinct compartments, each with differential inputs, gene expression, connectivity, and distributions of neurotransmitters and neuromodulators^[53-61]^. These two compartments are referred to as striosomes (also known as patches) and matrix. When considering striatal input from midbrain DANs, unique sets of DANs produce projections to the patch and matrix compartments in both rodents and primates^[62,63]^. This organization of SNc DAN projection begins during development. Early on, dopamine input to postnatal striatum is organized into patch compartments. As development progresses, the matrix is eventually innervated by its own DA afferents^[50,52,64,65]^.

Recent work has demonstrated that even within a specific target region, DAN subtypes have partially overlapping yet distinct projections^[66]^. With respect to SNc DANs, three projection patterns were identified. These projections segregate along different axes of the caudate putamen. One of these projection patterns projects for the most part to locomotor areas of the striatum and consists of neurons positive for ALDH1A1, Sox6, and Ndnf that mainly project their fibers to rostral, intermediate, and caudal caudate putamen^[66]^. Another projection group originating in the dorsal SNc consists of neurons positive for CALB1 and Sox6 but negative for ALDH1A1 that project to the medial rostral caudate putamen and ventromedial regions of the intermediate and caudal caudate putamen. A third group consists of neurons positive for Vglut2 and CALB1 but negative for Sox6 that are located in the lateral SNc and project to the tail of the caudate putamen more so in response to novel cues and salience than error prediction^[67]^. DANs that project to cortical and limbic areas in both rat and monkey, while mostly derived from the VTA, are also present in lower numbers in the dorsal tier of the SNc^[47,68-70]^.

In addition to projections, research has also investigated the inputs to nigrostriatal DANs and supports an integration of inputs from the autonomic, somatosensory, and motor areas. The greatest source of input is from the dorsal striatum, with a lot of input coming from the globus pallidus as well^[71]^. There are also notable projections from the central nucleus of the amygdala, entopeduncular nucleus, bed nucleus of stria terminalis, paraventricular hypothalamus nucleus, parasubthalamic nucleus, zona incerta, superior colliculus, supraoculomotor periaqueductal gray, dorsal raphe nucleus, pendunculotegmental nucleus, cuneiform nucleus, and parabrachial nucleus^[71]^. Additionally, strong excitatory inputs from the subthalamic nucleus and somatosensory and motor cortices may contribute to quick responses in the SNc DANs during salient events.

### Functional diversity of SNc DAN subtypes

Various genetic, behavioral, and pharmacological studies have established a role of the nigrostriatal dopamine pathway in motor function, with reward largely associated with the mesolimbic dopamine pathway, which consists of dopaminergic connections that project from the VTA to the ventral striatum^[49]^. However, research also supports the importance of the nigrostriatal dopamine pathway in reward, suggesting that both SNc and VTA midbrain DANs have altered firing in response to reward prediction and prediction errors^[72]^. Studies show that reward-predicting stimuli result in SN activation, highlighting the fact that changes in midbrain DAN activity are not limited to the VTA^[73]^. When considering SNc DANs specifically, there is again support for neuronal activation in response to reward or sensory stimuli that predict reward and inhibition by aversive stimuli^[74]^. These DANs are located within the ventromedial part of the SNc adjacent to VTA, which raises the need to better define those two DAN subpopulations with more definitive genetic makers. On the other hand, there are also some SNc DANs that are activated by aversive stimuli or cues that predict aversive stimuli, which are located in the dorsolateral part of the SNc^[74]^. The role of dopamine is expanding further to include response to novel, salient, and even aversive stimuli^[75-81]^.

The entire reward circuitry within a brain is a complex neuronal network with different aspects of reward- and incentive-based learning associated with pathways and connections within the larger reward network^[49]^. Reward systems are strongly associated with not only reward processing but also cognitive planning and motor control pathways that together all contribute to implementing an action plan for goal-directed behavior in response to reward and motivation^[49]^. For a long time, the basal ganglia were well known mostly for their role in motor behavior^[49]^. The nigrostriatal pathway has commonly been recognized as being involved in the facilitation and control of voluntary movement^[82]^. Nigrostriatal DANs allow information regarding movement to be sent from the SN to the striatum and are critical for normal movement capabilities. When information is transmitted to the striatum, desired movements can be initiated^[83]^. Nigrostriatal dopamine input to the striatum also plays a role in the initial learning and memory of sequential motor tasks and motor skill learning^[84,85]^. We now know that the basal ganglia are involved not just in motor function, but more widely in a range of emotional, cognitive, and motivation functions that allow for goal-directed behaviors^[49]^.

In terms of where in the SNc motivational value or salience signals, reward value coding, activity in response to aversive stimuli, and signals for trial start of unexpected time cues occur, each specific signal type is most strongly associated with some part within the SNc^[75]^. Ventromedial SNc DANs are strongly associated with motivational value signals, dorsolateral SNc DANs are strongly associated with motivational salience signals, ventromedial SNc DANs are strongly associated with standard reward value coding, lateral SNc DANs are strongly associated with aversive cues that lead to excitation, and DANs throughout the SNc are associated with trial start cues and unexpected time cues^[74,86,87]^. Adding to what we know about reward prediction error of dopamine signaling, recent research has worked on investigating the role of dopamine in impulsivity^[88-91]^. Different SNc groups and their corresponding projections are suggested to be able to distinguish between decisional impulsivity and motor impulsivity. Research suggests that the two types of impulsivity are regulated by different dopamine systems^[88]^. Specifically, the medial SNc dopamine group plays a role in value-coding, or the difference in response between reward and punishment, and is associated with impulsive choice. On the other hand, the ventral and lateral SNc DAN groups play a role in salience-coding, or the difference in response between either reward or punishment and aversive stimuli, which are associated with response inhibition.

Differences in function between ventrally and laterally positioned SNc DANs have also been demonstrated for other behaviors. ATP-sensitive potassium (K-ATP) channel activity in medial SN DANs, but not lateral SN DANs, allows for *in vivo* burst firing that is critical for novelty-dependent exploratory behavior but not standard locomotion^[92]^. Research also suggests nigrostriatal DAN activity signals the start or stop of action sequences and is involved in action selection, in addition to its role in reward-based learning^[93-96]^. Pathways that involve the SNc are also critical for learning to orient to food cues and for increasing motivation to perform reward-seeking actions^[75,97-101]^.

### Distinct characteristics of ALDH1A1-positive SNc DAN subpopulation

Although the classification of midbrain DANs into the A8, A9, and A10 cell groups is still commonly used, recent work suggests the existence and importance of functional and gene expression heterogeneity of subgroups within each of these cell groups^[12,14,66,75,102-105]^. One subtype of SNc DANs that is of particular interest, especially in the context of PD, is the ALDH1A1-positive subtype of SNc DANs^[106]^. ALDH1A1 oxidizes the highly reactive dopamine catabolite 3,4-dihydroxyphenylacetaldehyde (DOPAL)^[107]^, and neurons that express ALDH1A1 correspond to ventral tier nigrostriatal DANs, which are preferentially degenerated in PD^[15,38,108]^. The ALDH1A1-positive SNc DAN subtype accounts for about 70% of SNc DANs^[15,39]^. The ALDH1A1-positive subtype has its own pattern of projections and inputs that is distinct from the connectivity patterns of other SNc DAN subtypes, in addition to playing an important role in the acquisition of skilled movements in rodent models of PD^[39,106]^.

**Figure 2.**
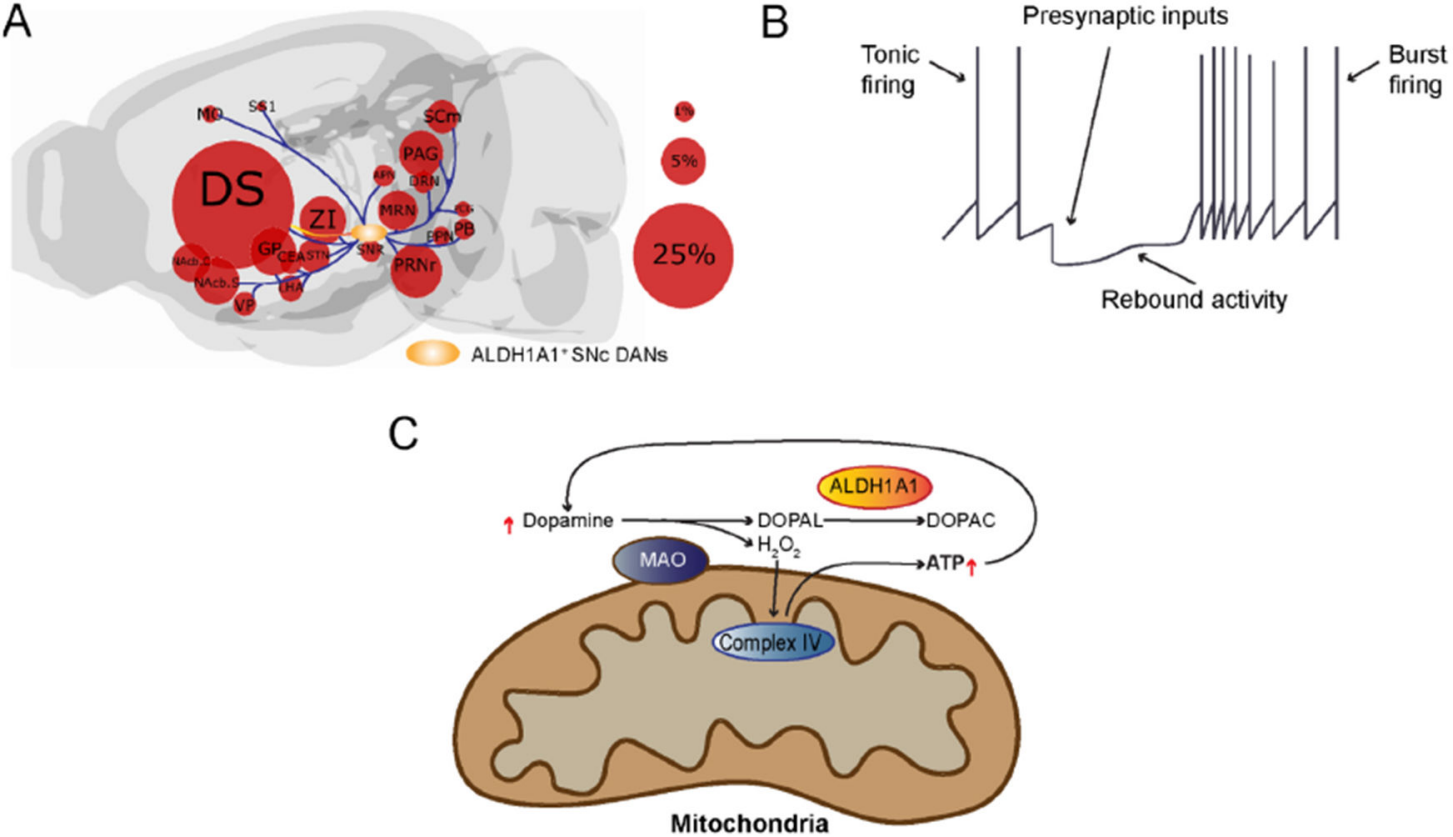
Presynaptic inputs alter the firing pattern of ALDH1A1-positive SNc DANs, in which the burst firing recruits dopamine for ATP production: (A) We outline the locations of major input neurons in the brain that directly innervate ALDH1A1-positive SNc DANs. While the ALDH1A1-positive SNc DANs integrate diverse synaptic inputs from different brain regions, they provide the output mainly to the dorsal striatum. The size of the circles represents the percentage of overall inputs. (B) Presynaptic inputs regulate the transition from tonic firing to burst firing of ALDH1A1-positive SNc DANs. (C) Cytosolic dopamine can be oxidized by mitochondria-attached MAO to produce H_2_O_2_ and DOPAL. H_2_O_2_ can be used for Complex IV-mediated ATP production, while the cytotoxic byproduct DOPAL can be neutralized by ALDH1A1. The increase of ATP production may lead to increased dopamine release and reuptake, resulting in further increase of ATP production and dopamine release during the burst firing. DS: Dorsal striatum; ZI: zona incerta; NAcb.C: nucleus accumbens core; NAcb.S: nucleus accumbens shell; MO: motor cortex; SS1: somatosensory cortex; VP: ventral pallidum; GPe: globus pallidus; STN: subthalamic nucleus; PAG: periaqueductal gray; APN: anterior pretectal nucleus; MRN: medial raphe nucleus; PRNr: pontine reticular nucleus roastral; PPN: posterior pretectal nucleus; PCG: pontine central gray; SCm: superior colliculus medial; DRN: dorsal raphe nucleus; CEA: central nucleus of the amygdala; LHA: lateral hypothalamus; PB: pontine parabrachial nucleus.

ALDH1A1-positive DANs in the SNc project primarily to the dorsal striatum^[39,109]^. The projections to the striatum appear to be arranged along a medial to lateral axis based on the position of their cell bodies. The more caudal ALDH1A1-positive SNc DANs project to more rostral areas in the striatum^[39]^. However, only a small fraction of ALDH1A1-positive SNc DAN axons converge to the striosomes^[39,66,110]^. In terms of inputs, ALDH1A1-positive SNc DANs receive most of their input from the caudate putamen, but they also receive substantial input from other areas in the striatum, pallidum, hypothalamus, and midbrain [Figure 2A]^[39]^. While there is less input from cortical areas, most of the input that is derived from there is coming from primary and secondary motor cortices and the somatosensory cortex, implicating a role of the regulation of ALDH1A1-positive SNc DANs in sensorimotor activity^[39]^. Relative to ALDH1A1-positive DANs in the VTA, those in the SNc receive more inputs from the caudate putamen, particularly the lateral caudate putamen which is heavily innervated by the ALDH1A1-positive SNc DANs, supporting a strong reciprocal innervation^[39]^. Compared to inputs to all SNc DANs, more inputs to ALDH1A1-positive SNc DANs come from the ventral striatum and the hypothalamus while fewer inputs originate from neurons in the cerebral cortex, pallidum, amygdala, and midbrain^[39,71]^.

With respect to its distinct functional role, ALDH1A1-positive SNc DANs are critical for the acquisition of motor skill learning in the rotarod task in a mouse model that is not alleviated by dopamine replacement therapy^[39]^. More generally, it seems that timely and dynamic regulation of dopamine release by ALDH1A1-positive SNc DANs plays an important role in mediating goal-oriented actions requiring high levels of motor motivation. Compared to the ALDH1A1-negative SNc DANs, the ALDH1A1-positive SNc DANs possess a distinct rebound activity after hyperpolarization [Figure 2B], resulting in alteration of firing pattern from evenly paced tonic firing to high frequency burst firing^[16,106,111]^. The burst firing may lead to increase of dopamine release, an indicator for engagement of certain actions. The presynaptic inhibitory inputs from striosome direct pathway spiny neurons play a major role in regulating the transition from tonic firing to burst firing of ALDH1A1-positive SNc DANs^[111]^. During the burst firing, the cytosolic dopamine can be oxidized by mitochondria-attached monoamine oxidase (MAO) to produce H_2_O_2_ and DOPAL [Figure 2C]^[112]^. The H_2_O_2_ can then be utilized for mitochondrial Complex IV-mediated ATP production^[112]^, while the increase of ATP production may lead to increased dopamine release and reuptake, resulting in further increase of ATP production and dopamine release during the burst firing [Figure 2C]. The presence of ALDH1A1 neutralizes the cytotoxic byproduct DOPAL and maintains the normal function and survival of this distinct DAN subtype^[106]^. However, there is still more research needed regarding the integration of specific excitatory and/or inhibitory inputs in functionally regulating ALDH1A1-positive SNc DANs before we fully understand the role of ALDH1A1-positive SNc DANs in motor learning.

## DIVERSE VTA DOPAMINERGIC NEURON SUBPOPULATIONS

VTA holds an intriguing and diverse population of DANs in the midbrain^[104]^. The VTA neurons synthesize several major neurotransmitters, including dopamine, GABA, and glutamate^[113]^. While most neurons in the VTA are dopaminergic, the exact percentage can vary between subregions. Overall, only around 50% exclusively secrete dopamine, while others co-secrete glutamate and GABA or do not secrete dopamine at all^[114,115]^. In contrast to the SNc, which is greatly associated with movement, the VTA is more related to emotion and cognition^[116,117]^. All of these functions are impaired in PD^[118]^, making both regions of great translational interest. The anatomical separation of the VTA is not clear, hence its name ending in “area”, not “nucleus”. Its separation from the SNc is best described based on both its functional projections and molecular markers^[104]^. While the SNc tends to project to the striatum via the nigrostriatal pathway, which is critical for motor movement, the VTA largely mediates dopamine secretion through limbic and cortical projections^[119]^. These two, divergent VTA pathways are known as the mesolimbic and mesocortical, respectively^[120]^. They are especially important for incentive-based behavior, motivation, and cognition^[121-123]^. The mesocortical pathway projects to the prefrontal cortex and is related to the attention to reward experience, interpretation of motivation, and the cognitive appraisal to seek out reward again^[122]^. On the other hand, the mesolimbic pathway projects to limbic structures, such as the amygdala, nucleus accumbens, and hippocampus^[124]^. Different stimuli can lead to different degrees of dopamine secretion or firing patterns^[125-127]^, helping to explain why some drugs may be more addictive than others.

### Molecular, anatomical, and functional subclassification of VTA components

The VTA DANs were originally classified into five subgroups but are presently further segregated into seven due to analyzing the differential expression of GIRK2, calbindin, DAT, and TH^[128]^: interfascicular nucleus (IF), rostral linear nucleus (RL), caudal linear nucleus of the raphe (CLi), paranigral nucleus (PN), parabrachial pigmented nucleus (PBP), parainterfascicular nucleus (PIF), and ventral tegmental area rostral (VTAR) [Figure 1]. The VTA has a greater diversity of DAN subtypes when compared to RRF and SNc groups. Despite these classifications, several studies have detected heterogeneity, even within the clusters, both molecularly and functionally^[129]^. Some of the main differences found were between the medial and lateral VTA DANs^[128,130]^. These regions tended to have more calbindin-positive DANs and less DAT- and GIRK2-positive expression; in addition, the IF subregion had the smallest size DANs out of all VTA nuclei^[128]^. Moreover, in the lateral VTA, PBP and VTAR are on the edge of the SNc but remain distinctly classified as VTA neurons based on molecular markers^[128]^. Compared to SNc neurons, the PBP has a greater ratio of calbindin/TH-positive neurons, with neurons immersed in fibers aligned in different directions^[128]^. Likewise, VTAR DANs are remarkably less densely packed, making them distinct from the SNc^[128]^. There have been attempts to better organize midbrain DAN clusters, such as with single cell expression profiling in neonatal brains^[129]^. Certain genes were enriched in the SNc or the VTA and were used to analyze their relative expression across clusters^[129]^. For instance, Otx2 is mostly expressed in VTA region, while SOX6 is mostly expressed in SNc^[129]^. Moreover, ALDH1A1 served as a useful distinguishing marker between clusters, related to how ALDH1A1 is anatomically mainly expressed in the ventral VTA and SNc^[129]^.

Much remains unknown about output projection patterns from the VTA, especially with regards to different DAN subpopulations^[131]^. An exception is the RLi subregion, which has been well studied with regards to outputs^[132]^. This is further complicated by the presence of co-secreting neurons, which may secrete any combination of dopamine, GABA, and glutamate^[133]^. Even more, certain neurons do not secrete or reuptake dopamine despite synthesizing it and expressing TH^[104]^. The different circuits and functions of these VTA DAN subtypes is still somewhat a mystery and is motivating further research on projection patterns. Furthermore, function and regulation of VTA DANs can be influenced by differential upstream groups of neurons to yield reward and aversion^[134,135]^. This highlights how complex inputs can be in regulating the already complex diversity of VTA DANs. Better determining their transcriptome, connectivity, and functionality may be useful in better classifying and understanding the very diverse populations of DANs in the VTA.

## DIVERSE SUSCEPTIBILITY OF DOPAMINERGIC NEURON SUBTYPES IN PARKINSON’S DISEASE

PD involves loss of both SNc and VTA DANs^[136]^; however, the contribution of DAN loss in the VTA to PD symptoms remains controversial among scientists and physicians alike. Results from a series of studies comparing DAN counts in SNc and VTA across PD and healthy control brains stained with TH demonstrate the involvement of the VTA in PD^[136]^. Although researchers observed significantly more degeneration of DANs within the SNc, the substantial neurodegeneration within the VTA may contribute to PD-related clinical symptoms, especially the non-motor syndrome^[136]^. It needs to be pointed out that in the previous studies the boundary between SNc and VTA was often drawn arbitrarily based on a few anatomical landmarks [Figure 1]. With the availability of increasing numbers of distinctive genetic markers, the function and survival of SNc and VTA DAN subtypes will be investigated in a more precise and molecularly defined way. In other words, future experiments are expected to pinpoint which DAN subtypes in the SNc and VTA DANs are involved, and the extent to which they are degenerated in PD. We suspect that the selective susceptibility of DAN subtypes during the progression of PD may contribute to the complex clinical manifestations of the disease.

### Diverse vulnerability of SNc DAN subtypes

A major pathological characteristic of PD, the major cause of parkinsonism, is the preferent neurodegeneration of SNc DANs. The loss of SNc DANs is associated with both bradykinesia and rigidity, two of the major motor symptoms that occur in PD^[137]^. As for the reason the SNc DANs are preferentially degenerated in PD, there are multiple distinct characteristics pertaining to endogenous neurotransmitter expression, structure, physiology, and local environmental conditions of the SNc DANs that may make them intrinsically vulnerable to degeneration^[138]^.

#### Dopamine as a neurotransmitter

The fact that these neurons are dopaminergic suggests that dopamine itself might play a role in contributing to selective vulnerability^[139]^. In many ways, the oxidative chemistry of dopamine can be associated with mechanisms underlying pathways involved with dysfunction of protein degradation, deficits in mitochondria processes, protein aggregation, neuroinflammation, and oxidative stress^[139,140]^. Free radicals and quinones that ultimately derive from the presence of dopamine can go on to interact with different cellular components and eventually contribute to the pathogenesis of PD. In the cytosol, dopamine is synthesized from tyrosine. Specifically, tyrosine is converted into L-dihydroxyphenylalanine (L-DOPA) by TH, the rate-limiting enzyme in dopamine synthesis, which is subsequently converted into dopamine by aromatic amino acid decarboxylase^[138,139]^. Dopamine is stabilized by the low pH within synaptic vesicles following its sequestration there by VMAT2^[141]^. Unlike when dopamine is within vesicles, it readily self-oxidizes in the cytosol, and too much oxidized dopamine is thought to be toxic to the DANs^[138]^. One consequence of oxidation is the formation of reactive oxygen species (ROS), which, when present at too high of levels, can damage DNA, RNA, proteins, and lipids^[142]^. Dopamine is also capable of being oxidized into reactive quinones (DAQs) that may also alter DNA, proteins, and lipids or form DNA-adducts leading to DNA damage responses^[143,144]^. Self-oxidation of dopamine within the neurons is supported by the presence of neuromelanin, as DAQs serve as precursors for two different portions of neuromelanin: pheomelanin (the polymeric core of neuromelanin) and eumelanin (the polymeric surface of neuromelanin)^[145,146]^. Although neuromelanin is believed to be non-toxic and maybe even neuroprotective^[147-149]^, neurons in the SNc with high levels of neuromelanin ultimately have the greatest vulnerability in PD^[25,150]^. This is consistent with the observation that neuromelanin can increase α-synuclein levels via inhibition of proteasomal degradation which ultimately contributes to Lewy body pathology^[145]^. α-synuclein is natively unfolded and will associate with vesicle membranes. It can also form into oligomers, also known as protofibrils, which are able to permeabilize dopamine-containing synaptic vesicles and cause dopamine to leak into the cytosol^[151]^. The cytosolic dopamine can then react with α-synuclein and create an adduct that slows the conversion of photofibrils into fibrils. This effectively maintains the presence of photofibrils, which causes more synaptic vesicle permeabilization and thus more dopamine leakage, creating a cycle of increasing cytosolic dopamine^[152-154]^. α-synuclein-dopamine adducts can also block chaperone-mediated autophagy by preventing lysosome receptors from accepting proteins and breaking them down, contributing to an increasing number of toxic proteins in the neuron^[155]^.

Dopamine in the cytosol can also be metabolized by MAO to form DOPAL, which is very toxic and can enhance α-synuclein aggregation^[156]^. To protect itself, neurons will attempt to condense oxidized products, turning them into neuromelanin^[147]^. MAO, in its metabolization of dopamine, has been thought to produce hydrogen peroxide in the cytosol^[112,157]^. Recent evidence in both mouse and human DANs, however, shows that dopamine metabolism by MAO does not increase hydrogen peroxide levels in the cytosol; instead, it increases mitochondrial electron transport chain activity^[112]^. Perhaps electrons generated from dopamine metabolism are not transferred to oxygen but rather brought through the mitochondrial intermembrane space to the electron transport chain^[112]^. Although there is certainly a lot of evidence suggesting a potential pathogenic role of dopamine in the SNc neurons, there are other neurons that do not express dopamine but are still degenerated to some degree in PD. There are also DANs in other areas of the brain that are not degenerated in PD^[138]^. This suggests that dopamine’s presence alone in SNc DANs is not the only factor contributing to the neurons’ intrinsic vulnerability.

#### Preferential degeneration of ALDH1A1-positive SNc DANs in PD

DANs in the ventral tier of SNc displayed the most profound loss in the postmortem brains of PD patients^[15,158]^. These ventral SNc DANs selectively express ALDH1A1^[15,38]^. As one of the 19 members of ALDH superfamily genes in the human genome^[159]^, ALDH1A1 is the only one exclusively expressed by the midbrain DANs^[15,38]^. Within DANs, ALDH1A1 converts the highly reactive dopamine catabolic intermediate cytotoxic DOPAL into a less toxic acid form and thereby protects DANs against DOPAL-induced cytotoxicity^[160]^. The generation of DOPAL used to be regarded as a passive event due to the oxidation of dopamine leaked in the cytosol^[38,161]^. A recent study suggests that the oxidation of cytosolic dopamine may actively participate oxidative phosphorylation and ATP production in DANs in response to intensive extracellular stimulations^[112]^. ALDH1A1 would be a key enzyme to neutralize the production of cytotoxic DOPAL during this process. The levels of ALDH1A1 expression are downregulated in PD^[15,162]^, while genetic ablation of ALDH1A1 and ALDH2 causes robust SNc DAN loss and motor impairments in aged mice^[163]^. Genetic variants in the ALDH1A1 gene locus were associated with sporadic PD cases^[164]^, while epidemiological studies link high exposure of fungicide benomyl, a potent ADLH inhibitor, to increased PD risk^[165]^. The reduction of ALDH1A1 expression may render the ventral SNc DANs more susceptible to cytosolic stresses^[38]^. ALDH1A1 expression level and activity can be used as an important biomarker to monitor the progression of the disease, while enhancement of ALDH1A1 activity could serve as a potential therapeutic strategy^[38]^.

#### Distinct neuronal architecture: long axonal arbor and lots of branches

The terminal field of SNc DANs are thought to be lost before cell bodies based on observations of human brains, suggesting this part of the SNc DANs is most vulnerable^[158,166-169]^. Multiple structural characteristics of the SNc DANs, specifically their terminal fields, may contribute to the selective vulnerability. The terminal fields of the SNc DANs are very large, dense, and wide and thus form a uniquely large number of synapses with the striatum and have many sites where neurotransmitter release can occur^[170-174]^. Additionally, the SNc DAN axons are long and unmyelinated with complex axonal arborization^[174]^. This creates a high energetic burden for action potential propagation which might contribute to increased basal stress on these neurons and make them more vulnerable to further environmental and genetic stressors^[172,174,175]^. Aside from long axons possibly contributing to high bioenergy requirements, the many dopamine release sites with their need for large vesicle pool and release machinery such as α-synuclein could also be predicted to be relatively energetically expensive^[138]^.

The energetically expensive physical maintenance of protein synthesis, cytoskeleton structure, membrane potential, and synaptic transmission of such a complex neuronal architecture as that of SNc DANs may also increase vulnerability^[138]^. Although high, the neurons are normally able to handle the energetic demand. However, it is when exposure to any of multiple possible environmental or genetic perturbations disrupt the link between energy production and demand that problems begin to occur^[174]^. All the necessary proteins, lipids, and organelles that are in the soma must be transported to distal sites via anterograde transport while some damaged structures need to be brought back to the soma for degradation via retrograde transport^[176-180]^. Importantly, all the transporting is happening within one long axon, which means the transported materials are much more likely to get crowded and experience disrupted trafficking^[177,178]^. Disrupted transport of mitochondria particularly would severely limit the spatial distribution of mitochondria, which serve as the main energy source of neurons, and thus the spatial distribution of energy sources within the neuron. Ultimately, it seems that the long and highly branched axons of SNc DANs are particularly susceptible to the disruption of mitochondria dynamics, making these neurons particularly prone to axon degeneration.

#### Autonomous firing

Beyond distinct structural properties, SNc DANs also exhibit unique physiological characteristics that may contribute to increased and selective vulnerability to degeneration. One such characteristic is that the neurons are slow, autonomous pacemakers (unlike most neurons in the brain) with broad action potentials due to Ca^2+^ influx^[181]^. This means that the SNc DANs will spike, resulting in Ca^2+^ entry into the cell, despite receiving no excitatory input^[182-188]^. Autonomous firing and subsequent Ca^2+^ entry into the neuron create an energy demand. Additionally, the continuous firing and broad action potentials cause the ionic gradients underlying excitability to be eliminated^[138]^. To maintain an electrochemical gradient, ATP-dependent pumps must be maintained, contributing further to energy demand. The neurons will also respond to synaptic inputs which can trigger burst firing and further increase Ca^2+^ loading within the neurons^[189,190]^.

SNc DANs express low-threshold variants of the CaV1.3 L-type calcium channel, unlike most other pacemaker neurons. There is a sustained Ca^2+^ influx because these channels never fully close^[186,187]^. Ca^2+^ in the cytosol easily crosses the outer membrane of mitochondria via large nonselective pores; crossing the inner membrane of the mitochondria, however, is tightly regulated. The mitochondrial Ca^2+^ uniporter is a selective ion channel that allows Ca^2+^ to enter the matrix^[191,192]^. Ca^2+^ within the mitochondria increases tricarboxylic acid enzyme activity and oxidative phosphorylation^[193-195]^ which will eventually lead to ROS^[140,181]^. Any generated oxidants that are not taken care of by antioxidant defenses can cause a continuous oxidative stress in the mitochondria^[196,197]^. Continuous oxidative stress in the mitochondria can have many negative effects, including increased sensitivity of SNc DANs to toxins and ageing^[181,198,199]^.

Sustained levels of Ca^2+^ that remain in the cytosol can also have negative effects, specifically by leading to increased α-synuclein accumulation^[200-202]^. The high levels of Ca^2+^ in the SNc DANs is not buffered much by calbindin^[203]^. Without sufficient buffering, Ca^2+^ can diffuse away to various targets within the cell. Mutant α-synuclein is able to increase pacemaker activity (and the subsequent stress it causes) by disrupting A-type K^+^ channel^[204]^. Supporting the intrinsic vulnerability of the SNc DANs, this occurs in SNc but not VTA DANs.

#### Synaptic partners

The unique cellular environment and local neurons present near the SNc DANs may also contribute to their distinct vulnerability to neurodegeneration. Research now supports the concept that SNc DANs are relatively depolarized under normal, healthy conditions. The membrane potential of the neurons usually sits between −60 and −45 mV^[205,206]^. At this potential, the Mg^2+^ block of N-Methyl-D-aspartate receptors (NMDARs) is relatively weak. Additionally, for the most part, SNc DANs express NMDARs containing the GluN2D subunit, which is relatively insensitive to Mg^2+[206,207]^. Together these observations suggest that there are lots of NMDAR openings at any given time, even in healthy neurons, which can increase Ca^2+^ loading and oxidant stress in the SNc DANs. Upon prolonged exposure to high levels of glutamate outside the SNc DANs, metabotropic glutamate receptors (mGluRs) are also activated. Via a process referred to as Ca^2+^-induced Ca^2+^ release, activation of both mGluRs and NMDARs prompt the endoplasmic reticulum to release Ca^2+[208]^.

Additionally, the SNc DANs are surrounded by a relatively high density of microglia, which are involved in inflammatory responses^[209]^. Microglia can become activated when exposed to proinflammatory molecules, toxins, and protein aggregates, prompting the microglia to then release molecules that may be harmful to the neurons^[210-213]^.

### Involvement of VTA DAN subtypes in PD-related non-motor symptoms

Abnormal prefrontal dopaminergic and cholinergic circuits lead to a variety of cognitive symptoms, including executive dysfunction, hallucinations, and psychosis^[214]^. Further, the development of cognitive symptoms may serve as a predictor for PDD and amnestic dysfunction as the disease progresses^[214]^. To examine the neurophysiological underpinnings of PD-related depression, researchers lesioned both the SNc and VTA of rats and measured the effect of L-DOPA and citalopram administration, an amino acid precursor to dopamine and a selective serotonin reuptake inhibitor, respectively^[215]^. While depressive-like behavior was induced by lesioning either the SNc or VTA, symptoms were alleviated by either drug treatment^[215]^. These results suggest a link between both SNc and VTA DAN deficits with PD-related depression, as well as the involvement of serotonergic pathways^[215]^. Another study found that partial bilateral ablation of the SNc results in both motor and non-motor symptoms, while ablation of both the SNc and the tail of the VTA relieves symptoms in PD mouse models^[216]^. These data demonstrate the compensatory role of the VTA in moderating the DA system in response to SNc neuronal ablation^[216]^. Although researchers tend to focus on the involvement of the SNc due to severe neurodegeneration of DANs localized in this brain region, these findings support the role of the VTA in PD symptoms^[217]^.

#### VTA DAN subpopulations in the formation of declarative memory and PDD

Accumulative evidence supports an association of dopaminergic dysfunction with PDD^[117,218]^. PDD is likely resulted from extensive degeneration of midbrain DANs beyond the SNc regions in the late stages of PD. Which subpopulations of midbrain DANs contribute to PDD remains to be determined. With the advancement of gene profiling in individual neurons, many genetically defined DAN subtypes have been identified in different SNc and VTA subregions^[14,17,43]^. Using an intersectional genetic labeling strategy, a recent study found that a cluster of vesicular glutamate transporter 2-positive (VGLT2^+^) DANs in the ventral VTA project predominantly to the entorhinal (ENT) and prefrontal cortices^[66]^. Interestingly, ENT atrophy is particularly associated with PDD^[219]^. By contrast, VTA DANs only sparsely project to the hippocampal formation^[66]^. Instead, the hippocampus receives the most dopamine inputs from the afferent fibers of locus coeruleus^[220]^. Therefore, it would be interesting to investigate the synaptic inputs and physiological functions of VTA-VGLT2^+^ DAN subpopulations in declarative memory formation. The knowledge gained from this study will provide cell type and circuit specific mechanisms of PDD and lay the foundation for designing new therapeutic interventions for treatment of cognitive impairments in PDD.

#### VTA DAN subpopulations in temporal control of movement and PD

Past work indicates the role of VTA in temporal control, or “guiding movements in time to achieve behavioral goals”^[221]^, as well as temporal expectation, or “the ability to anticipate when a stimulus occurs in time”^[222]^. Researchers experimentally manipulated prefrontal dopamine transmission from the VTA of rodents to examine the effect on temporal control during a fixed-interval task^[221]^. Temporal control was impaired by viral RNA interference with VTA DA transmission, antagonists blocking dopamine receptor D1 (DRD1) in the medial prefrontal cortex, and optogenetic inhibition of prefrontal DRD1-positive neurons^[221]^. Further, temporal control during the fixed-interval task was improved by optogenetic stimulation of prefrontal DRD1-positive neurons^[221]^. These results suggest the involvement of mesocortical DAN projections and DRD1 within prefrontal cortex over temporal control of movement^[221]^. Another study investigated temporal expectation during a reaction time task in dopamine-depleted rats, while inhibiting DAN projections from the VTA with a selective neurotoxin^[222]^. Although VTA dopamine depletion did not alter movement and learning during the reaction time task, rats did not exhibit delay-dependent speeding^[222]^. These data suggest the involvement of mesocortical DAN circuits in temporal expectation^[222]^. Researchers also found that delay-dependent speeding was reduced by DRD1 antagonist but not DRD2 antagonists, indicating the role of prefrontal cortex DRD1 in temporal expectation^[222]^. Taken together, these studies suggest that VTA function significantly impacts temporal control and expectation, specifically highlighting the role of DRD1 subtype in animal models of PD.

## DIVERSITY OF MIDBRAIN DOPAMINERGIC NEURON SUBTYPES IN NORMAL AGEING

Normal ageing is associated with minor neurodegeneration of DANs within the SNc, accompanied by a significant decline in voluntary motor control^[223]^. Brain imaging studies indicate a significant loss of DAN function due to normal ageing, linking deficits in DA neurotransmission to cognitive dysfunction^[224]^. Although dopamine neurotransmission may impact cognitive performance directly, it may also act in an age-dependent manner^[224]^. Normal ageing has been associated with impaired episodic memory, processing speed, and executive functioning^[224]^. While DAN degeneration is experienced in both PD and normal ageing, loss of DAN function occurs at a more rapid rate in PD relative to healthy elderly subjects^[225]^. Despite differential changes in the SNc, links between DAN degeneration and cognitive dysfunction are common across both groups^[225]^. Further, research suggests that gonadal hormones modulate DA pathways, with sex differences in DANs and disease progression of PD and dementia^[226]^. Researchers examined the effect of estrogen on SNc DANs in African green monkeys, finding that 30 days of estrogen deprivation led to permanent loss of more than 30% of DANs within the SNs^[226]^. Subsequent estrogen replacement only restored TH-immunoreactive cells when given 10 days after ovariotomy, but not 30 days after^[226]^. Taken together, research indicates demographic factors that may influence the degree and rate of cognitive decline experienced in PD, including both age and sex.

Past work suggests that dopamine plays a significant role in motivated behavior, such that DANs and cognitive function simultaneously decline in an age-dependent manner^[227]^. L-DOPA treatment has successfully enhanced reinforcement learning in elderly subjects, suggesting a link between DAN activity and motivated behavior in both PD and normal ageing^[227]^. Ageing also affects the progression of PD, including the age of symptom onset as well as the form and severity of PDD^[228]^. One longitudinal study found that the average PD patient experiences rapid DAN degeneration within the midbrain and progressive accumulation of Lewy bodies, which eventually invade the neocortex and cause PDD^[228]^. However, late-onset PD patients experience greater levels of Lewy bodies containing α-synuclein in addition to plaque formation, having a shorter disease course^[228]^. These data indicate how normal ageing may modulate the relationship between the DAN degeneration and decline in cognitive function.

### Ageing-related mitochondrial dysfunction in DANs

Mitochondrial dysfunction may play a role in the age-dependent mechanisms underlying DAN deficits in the SNc, such that mitochondrial DNA mutations increase with age^[223]^. For example, human studies indicate high levels of deletions in mitochondrial DNA of both PD and healthy elderly subjects, linking mitochondrial dysfunction with number of deletions^[223]^. Researchers also found a 20% increase in mitochondrial DNA deletions in old mice relative to young mice, also working in an age-dependent manner^[223]^.

### Ageing-related nitrative stress in DANs

Ageing serves as the highest risk factor for the development of PD, with nitrative stress potentially contributing to degeneration of the DA system^[229]^. Researchers assessed the link between nitrative damage and DAT levels in rhesus monkeys over time^[229]^. The number of DANs that underwent nitrative damage significantly increased with ageing in the SNc but not in the VTA. Further, the percentage of DANs that underwent nitrative damage was significantly higher in the SNc relative to the VTA^[229]^. These results demonstrate the age-dependent accumulation of nitrative damage and its role in selective neurodegeneration of the SNc DA system^[229]^.

### Ageing-related dysfunction of dopamine reuptake in DANs

DAT mediates the reuptake of dopamine from extracellular space into DANs^[230]^. One study assessed whether a decline in DAT expression was responsible for functional differences in DAT^[231]^. Although DAT immunoreactivity within the striatum, SNc, and VTA was not altered in an age-dependent manner, a 60% decrease of VTA TH was recorded only in older rats^[231]^. Further, a 30% decrease in dopamine reuptake and DAT protein recovery was recorded in the striatal synaptosomes of old rats relative to young rats^[231]^. These results indicate that reduced DAT expression on the plasma membrane results in age-related decline in DAT function^[231]^. Taken together, past work has established a robust relationship between ageing and DAT function in animal models of PD.

Positron emission tomography has been used to compare DAT levels across PD and healthy control subjects over time^[232]^. One longitudinal study found lower baseline DAT expression in PD relative to healthy controls, with a difference of 5.5% in the ventral striatum, 26.2% in the pre-commissural dorsal caudate, 29.9% in the post-commissural dorsal putamen, 34.5% in the pre-commissural dorsal putamen, and 60.2% in the post-commissural putamen^[232]^. Further, in each region of interest, the annual rates of DAT decline were 5.3%, 5.4%, 8.5%, 6.2%, and 7.8%, respectively^[232]^. This exponential pattern of DAT reduction demonstrates the normal ageing effect in PD^[232]^. Another study assessed age-related DAT decline in relation to motor function in normal ageing^[233]^. Although binding potentials of the DAT marker did not vary with age, researchers observed an inverse relationship between the marker for VMAT2 and age^[233]^. When split into age groups, performance on the motor task positively correlated with age in the younger group and negatively correlated with age in the older group^[233]^. These results suggest that age-dependent changes in VMAT2 and DAT act independently of one another, and that older individuals experience deficits in motor performance due to a decline in DAT binding^[233]^. Studies employing positron emission tomography offer further support for the relationship between normal ageing and DAT function, such that the age-dependent decline in DAT availability and binding contributes to impaired motor function in PD.

### Ageing-related changes of SNc DAN activity

Results from patch-clamp electrophysiological recordings suggest that DANs within the SNc have similar membrane capacitance and input resistance across age groups^[234]^. However, ageing leads to slower firing rates, narrower spike widths, variable interspace intervals, and smaller L-type calcium channel currents^[234]^. Therefore, normal ageing negatively impacts DAN function, impairing voluntary movement among other behavioral processes controlled by DA pathways^[234]^. Further, vulnerability of DANs within the SNc may be linked to progressive overreliance on L-type calcium channels with normal ageing^[235]^. This overreliance serves as a chronic stressor on the mitochondrial ATP that drives oxidative phosphorylation, resulting in neurodegeneration^[235]^. One study employed isradipine treatment to block L-type calcium channels, successfully reversing age-related overreliance on these channels^[235]^. Therefore, both functional changes in SNc DANs as well as overreliance on L-type calcium channels occur with normal ageing, resulting in neurodegeneration and motor dysfunction.

Evidence supports the accumulation of various pathological changes in normal postmortem SNc neurons compared to neurons from other brain regions of the same age. Such changes include mitochondria dysfunction, increased protein oxidation, higher levels of astrocytic proliferation, diminished antioxidant function, enhanced oxidative stress, neuromelanin accumulation, inability of neurons to appropriately handle calcium, and increased iron levels^[236-238]^. The various changes associated with ageing make the nigrostriatal DANs vulnerable to degeneration, and when combined with additional pathologies may ultimately lead to PD^[237]^. Studies across many animal models have investigated how the multiple processes associated with ageing effect the function and survival of SNc DANs, predisposing them to neurodegeneration. In wildtype mice, ageing alone leads to motor deficits, diminished striatal dopamine levels, fewer DANs, and fragmented mitochondria in DANs^[239]^. Research with rats have shown that mitochondrial DNA deletions in nigrostriatal DANs increase with age^[223]^. Studies investigating TH- and neuromelanin-containing DANs in non-human primates show that increasing age is associated with loss of neurons that only contain TH and an increase in neurons that only contain neuromelanin^[240]^. Age was also associated with loss of dopamine transporter-immunoreactive SN neurons^[241]^. These changes contribute to functional deficits by reducing striatal dopamine levels in older monkeys^[240,241]^. Cell vulnerability in response to injury [specifically, 1-methyl-4-phenyl-1,2,3,6-tetrahydropyridine (MPTP)] was also shown to increase with age^[240]^.

The effects of ageing on SNc DANs have also been linked to the diminished expression of specific genes. For example, the importance of *Nurr1* as ageing occurs has been demonstrated in mice^[242]^. Aged heterozygous *Nurr1*-deficient (*Nurr1*^+/−^) mice showed deficits in rotarod performance and locomotion that were associated with lower levels of striatal dopamine, less nigrostriatal DANs, and less *Nurr1* and DAT expression in the SN compared to wild-type controls. These results suggest an important role of Nurr1 in maintaining nigrostriatal DAN function and survival as ageing occurs. Studies in humans also suggest an important role of Nurr1. The transcription factor Nurr1 is important for establishing and maintaining dopamine phenotypes within the nigrostriatal DANs, and its diminished expression throughout ageing is associated with decreases in TH-positive neurons^[243-245]^. In PD, SNc DANs with decreased Nurr1 levels were also associated with increases in α-synuclein inclusions^[243]^. Together, these studies in humans and non-human primates suggests that ageing is associated with a downregulation of genes involved in dopamine transmission^[47]^.

Ageing is also associated with a decrease in the number of DAT-positive SNc neurons^[246]^. Comparing neurons from young (0-49 years), middle aged (50-69 years), and elderly (70-85 years) human samples show that, by middle age, the number of intensely stained DAT nigrostriatal neurons decreased while the number of lightly stained DAT nigrostriatal neurons increased. Increasing age is also associated with an increase in the number of cell bodies negative for DAT but positive for neuromelanin. Overall, each decade older was associated with a 6.7% decrease in the total number of nigrostriatal DANs.

### Ageing-related changes on the function and survival of VTA DANs

Interestingly, motivation has shown to be critical in age-related functional decline of the VTA^[247]^. In contrast to resting state contexts, VTA and ventral striatum functional coupling was enhanced in adolescence and decreased in adulthood in a motivational context, suggesting a distinguishing ageing marker^[247]^. Moreover, a decline of DAN function has been behaviorally associated with deficits in learning^[248]^. In terms of electrical physiology, the frequency of burst events of VTA DANs did not change with age, but bursts were longer in adolescents than in adults, potentially because GABA tone increases as rats reach adulthood. The firing rate increasing in adolescence is consistent with it being a more vulnerable time for developing drug addiction.

There are sex differences in functional connectivity of the VTA. For instance, men have a stronger VTA/SNc connectivity to the left posterior orbital gyrus than woman according to a study that measured resting state blood oxygenation level dependent signals^[249]^. Moreover, only men showed age-related functional VTA changes to cortical and cerebellar regions, implying that ageing differentially affects not only sexes but also distinct cerebral projections^[249]^. In a study on human post-mortem brain samples, there was no statistically significant loss of VTA DANs as a function of age, suggesting that the neurodegeneration implicated in ageing and PD is a result of but not the initiating cause of neuronal death^[250]^. However, more studies will be required to critically evaluate the function and survival of different VTA DAN subtypes during the normal ageing process.

## CONCLUSION

In the past decades, tremendous progress has been made in understanding the neurological and genetic causes of PD-related motor and non-motor impairments^[2]^, as well as how the different facets of the ageing process contribute to the progressive dysfunction and loss of DANs. However, due to a lack of distinctive molecular markers, the SNc or VTA DAN subpopulations were often studied as a homogenous unit, although many of these neuron subtypes display distinct connectivity, functionality, and susceptibility to ageing and PD. With the advance of single cell RNA sequencing technology, increasing numbers of molecularly defined midbrain DAN subtypes have been identified^[35]^. By employing intersectional genetic approaches, recent studies managed to genetically distinguish different midbrain DAN subpopulations. Various live imaging techniques with different genetically encoded sensors make it possible to directly correlate the neuron activity with behaviors and longitudinally monitor the neuron activity during ageing. The development of CRISPR/Cas9 gene editing, optogenetics, and chemogenetics procedures allow researchers to establish a causal relationship between neural activity and behavioral performance through genetically and functionally manipulating the neural activity. The knowledge gained from these ongoing studies may explain how different subtypes of DANs contribute to different aspects of behavioral phenotypes and provide new mechanistic insights into novel procedures for reconfiguring PD-induced behavioral abnormalities.

## References

[1] Parkinson J An essay on the shaking palsy. Sherwood, Nelly and Jones 1817.

[2] Lees AJ, Hardy J, Revesz T. Parkinson’s disease. Lancet 2009;373:2055–66. 19524782 10.1016/S0140-6736(09)60492-X

[3] Mhyre TR, Boyd JT, Hamill RW, Maguire-Zeiss KA. Parkinson’s disease. Subcell Biochem 2012;65:389–455. 23225012 10.1007/978-94-007-5416-4_16 PMC4372387

[4] Vijayakumar D, Jankovic J. Drug-induced dyskinesia, Part 1: treatment of levodopa-induced dyskinesia. Drugs 2016;76:759–777. 27091215 10.1007/s40265-016-0566-3

[5] Chaudhuri KR, Healy DG, Schapira AH; National Institute for Clinical E. Non-motor symptoms of Parkinson’s disease: diagnosis and management. Lancet Neurol 2006;5:235–45. 16488379 10.1016/S1474-4422(06)70373-8

[6] Emre M, Aarsland D, Brown R, Clinical diagnostic criteria for dementia associated with Parkinson’s disease. Mov Disord 2007;22:1689–707; quiz 1837. 17542011 10.1002/mds.21507

[7] Emre M Dementia associated with Parkinson’s disease. Lancet Neurol 2003;2:229–37. 12849211 10.1016/s1474-4422(03)00351-x

[8] Lopez-Otin C, Blasco MA, Partridge L, Serrano M, Kroemer G. The hallmarks of aging. Cell 2013;153:1194–217. 23746838 10.1016/j.cell.2013.05.039 PMC3836174

[9] Betarbet R, Sherer TB, MacKenzie G, Garcia-Osuna M, Panov AV, Greenamyre JT. Chronic systemic pesticide exposure reproduces features of Parkinson’s disease. Nat Neurosci 2000;3:1301–6. 11100151 10.1038/81834

[10] Hernandez DG, Reed X, Singleton AB. Genetics in Parkinson disease: Mendelian versus non-Mendelian inheritance. J Neurochem 2016;139 Suppl 1:59–74. 27090875 10.1111/jnc.13593 PMC5155439

[11] Blauwendraat C, Nalls MA, Singleton AB. The genetic architecture of Parkinson’s disease. Lancet Neurol 2020;19:170–8. 31521533 10.1016/S1474-4422(19)30287-X PMC8972299

[12] Lerner TN, Shilyansky C, Davidson TJ, Intact-brain analyses reveal distinct information carried by SNc dopamine subcircuits. Cell 2015;162:635–47. 26232229 10.1016/j.cell.2015.07.014 PMC4790813

[13] Menegas W, Bergan JF, Ogawa SK, Dopamine neurons projecting to the posterior striatum form an anatomically distinct subclass. Elife 2015;4:e10032. 26322384 10.7554/eLife.10032 PMC4598831

[14] Poulin JF, Zou J, Drouin-Ouellet J, Kim KY, Cicchetti F, Awatramani RB. Defining midbrain dopaminergic neuron diversity by single-cell gene expression profiling. Cell Rep 2014;9:930–43. 25437550 10.1016/j.celrep.2014.10.008 PMC4251558

[15] Liu G, Yu J, Ding J, Aldehyde dehydrogenase 1 defines and protects a nigrostriatal dopaminergic neuron subpopulation. J Clin Invest 2014;124:3032–46. 24865427 10.1172/JCI72176 PMC4071380

[16] Evans RC, Zhu M, Khaliq ZM. Dopamine inhibition differentially controls excitability of substantia nigra dopamine neuron subpopulations through T-type calcium channels. J Neurosci 2017;37:3704–20. 28264982 10.1523/JNEUROSCI.0117-17.2017 PMC5373143

[17] Hook PW, McClymont SA, Cannon GH, Single-cell RNA-Seq of mouse dopaminergic neurons informs candidate gene selection for sporadic Parkinson disease. Am J Hum Genet 2018;102:427–46. 29499164 10.1016/j.ajhg.2018.02.001 PMC5985341

[18] Przedborski S The two-century journey of Parkinson disease research. Nat Rev Neurosci 2017;18:251–9. 28303016 10.1038/nrn.2017.25

[19] Sveinbjornsdottir S The clinical symptoms of Parkinson’s disease. J Neurochem 2016;139 Suppl 1:318–24. 27401947 10.1111/jnc.13691

[20] Gershman SJ, Uchida N. Believing in dopamine. Nat Rev Neurosci 2019;20:703–14. 31570826 10.1038/s41583-019-0220-7 PMC7472313

[21] Albin RL, Young AB, Penney JB. The functional anatomy of basal ganglia disorders. Trends Neurosci 1989;12:366–75. 2479133 10.1016/0166-2236(89)90074-x

[22] Nelson AB, Kreitzer AC. Reassessing models of basal ganglia function and dysfunction. Annu Rev Neurosci 2014;37:117–35. 25032493 10.1146/annurev-neuro-071013-013916 PMC4416475

[23] Bentivoglio M, Morelli M. The organization and circuits of mesencephalic dopaminergic neurons and the distribution of dopamine receptors in the brain. Dopamine 2005;21:1–107.

[24] Weisenhorn DM, Giesert F, Wurst W. Diversity matters - heterogeneity of dopaminergic neurons in the ventral mesencephalon and its relation to Parkinson’s Disease. J Neurochem 2016;139 Suppl 1:8–26. 27206718 10.1111/jnc.13670 PMC5096020

[25] Hirsch E, Graybiel AM, Agid YA. Melanized dopaminergic neurons are differentially susceptible to degeneration in Parkinson’s disease. Nature 1988;334:345–8. 2899295 10.1038/334345a0

[26] Damier P, Hirsch EC, Agid Y, Graybiel AM. The substantia nigra of the human brain. II. Patterns of loss of dopamine-containing neurons in Parkinson’s disease. Brain 1999;122 (Pt 8):1437–48. 10430830 10.1093/brain/122.8.1437

[27] Hegarty SV, Sullivan AM, O’Keeffe GW. Midbrain dopaminergic neurons: a review of the molecular circuitry that regulates their development. Dev Biol 2013;379:123–38. 23603197 10.1016/j.ydbio.2013.04.014

[28] Smidt MP, Burbach JP. Terminal differentiation ofmesodiencephalic dopaminergic neurons: the role of Nurr1 and Pitx3. Adv Exp Med Biol 2009;651:47–57. 19731549

[29] Smidt MP. Molecular programming of mesodiencephalic dopaminergic neuronal subsets. Front Neuroanat 2017;11:59. 28769772 10.3389/fnana.2017.00059 PMC5515899

[30] Schein JC, Hunter DD, Roffler-Tarlov S. Girk2 expression in the ventral midbrain, cerebellum, and olfactory bulb and its relationship to the murine mutation weaver. Dev Biol 1998;204:432–50. 9882481 10.1006/dbio.1998.9076

[31] Karschin C, Dissmann E, Stuhmer W, Karschin A. IRK(1-3) and GIRK(1-4) inwardly rectifying K+ channel mRNAs are differentially expressed in the adult rat brain. J Neurosci 1996;16:3559–70. 8642402 10.1523/JNEUROSCI.16-11-03559.1996 PMC6578832

[32] Parent A, Fortin M, Cote PY, Cicchetti F. Calcium-binding proteins in primate basal ganglia. Neurosci Res 1996;25:309–34. 8866512 10.1016/0168-0102(96)01065-6

[33] Liang CL, Sinton CM, Sonsalla PK, German DC. Midbrain dopaminergic neurons in the mouse that contain calbindin-D28k exhibit reduced vulnerability to MPTP-induced neurodegeneration. Neurodegeneration 1996;5:313–8. 9117542 10.1006/neur.1996.0042

[34] Yamada T, McGeer PL, Baimbridge KG, McGeer EG. Relative sparing in Parkinson’s disease of substantia nigra dopamine neurons containing calbindin-D28K. Brain Res 1990;526:303–7. 2257487 10.1016/0006-8993(90)91236-a

[35] Poulin JF, Gaertner Z, Moreno-Ramos OA, Awatramani R. Classification of midbrain dopamine neurons using single-cell gene expression profiling approaches. Trends Neurosci 2020;43:155–69. 32101709 10.1016/j.tins.2020.01.004 PMC7285906

[36] Greene JG, Dingledine R, Greenamyre JT. Gene expression profiling of rat midbrain dopamine neurons: implications for selective vulnerability in parkinsonism. Neurobiol Dis 2005;18:19–31. 15649693 10.1016/j.nbd.2004.10.003

[37] Chung CY, Seo H, Sonntag KC, Brooks A, Lin L, Isacson O. Cell type-specific gene expression of midbrain dopaminergic neurons reveals molecules involved in their vulnerability and protection. Hum Mol Genet 2005;14:1709–25. 15888489 10.1093/hmg/ddi178 PMC2674782

[38] Cai H, Liu G, Sun L, Ding J. Aldehyde Dehydrogenase 1 making molecular inroads into the differential vulnerability of nigrostriatal dopaminergic neuron subtypes in Parkinson’s disease. Transl Neurodegener 2014;3:27. 25705376 10.1186/2047-9158-3-27 PMC4334846

[39] Wu J, Kung J, Dong J, Distinct connectivity and functionality of aldehyde dehydrogenase 1a1-Positive nigrostriatal dopaminergic neurons in motor learning. Cell Rep 2019;28:1167–81.e1167. 31365862 10.1016/j.celrep.2019.06.095 PMC6741442

[40] Tasic B Single cell transcriptomics in neuroscience: cell classification and beyond. Curr Opin Neurobiol 2018;50:242–9. 29738987 10.1016/j.conb.2018.04.021

[41] Zeng H, Sanes JR. Neuronal cell-type classification: challenges, opportunities and the path forward. Nat Rev Neurosci 2017;18:530–46. 28775344 10.1038/nrn.2017.85

[42] Poulin JF, Tasic B, Hjerling-Leffler J, Trimarchi JM, Awatramani R. Disentangling neural cell diversity using single-cell transcriptomics. Nat Neurosci 2016;19:1131–1141. 27571192 10.1038/nn.4366

[43] La Manno G, Gyllborg D, Codeluppi S, Molecular diversity of midbrain development in mouse, human, and stem cells. Cell 2016;167:566–80.e519. 27716510 10.1016/j.cell.2016.09.027 PMC5055122

[44] Tiklova K, Bjorklund AK, Lahti L, Single-cell RNA sequencing reveals midbrain dopamine neuron diversity emerging during mouse brain development. Nat Commun 2019;10:581. 30718509 10.1038/s41467-019-08453-1 PMC6362095

[45] Saunders A, Macosko EZ, Wysoker A, Molecular diversity and specializations among the cells of the adult mouse brain. Cell 2018;174:1015–30.e1016. 30096299 10.1016/j.cell.2018.07.028 PMC6447408

[46] Kramer DJ, Risso D, Kosillo P, Ngai J, Bateup HS. Combinatorial expression of Grp and Neurod6 defines dopamine neuron populations with distinct projection patterns and disease vulnerability. eNeuro 2018;5:ENEURO.0152-18.2018. 10.1523/ENEURO.0152-18.2018 PMC6104179 30135866

[47] Björklund A, Dunnett SB. Dopamine neuron systems in the brain: an update. Trends Neurosci 2007;30:194–202. 17408759 10.1016/j.tins.2007.03.006

[48] Haber SN. The place of dopamine in the cortico-basal ganglia circuit. Neuroscience 2014;282:248–57. 25445194 10.1016/j.neuroscience.2014.10.008 PMC5484174

[49] Haber SN, Knutson B. The reward circuit: linking primate anatomy and human imaging. Neuropsychopharmacology 2010;35:4–26. 19812543 10.1038/npp.2009.129 PMC3055449

[50] Gerfen CR, Herkenham M, Thibault J. The neostriatal mosaic: II. Patch- and matrix-directed mesostriatal dopaminergic and non-dopaminergic systems. J Neurosci 1987;7:3915–34. 2891799 10.1523/JNEUROSCI.07-12-03915.1987 PMC6569093

[51] Smith Y, Masilamoni J. The Substantia Nigra. Reference Module in Neuroscience and Biobehavioral Psychology. Elsevier; 2017.

[52] Gerfen CR, Bolam JP. The Neuroanatomical Organization of the Basal Ganglia. Handbook of Basal Ganglia Structure and Function. Elsevier; 2010. p. 3–28.

[53] Brimblecombe KR, Cragg SJ. The Striosome and matrix compartments of the striatum: a path through the labyrinth from neurochemistry toward function. ACS Chem Neurosci 2017;8:235–42. 27977131 10.1021/acschemneuro.6b00333

[54] Graybiel AM. Neurotransmitters and neuromodulators in the basal ganglia. Trends Neurosci 1990;13:244–54. 1695398 10.1016/0166-2236(90)90104-i

[55] Gerfen CR. The neostriatal mosaic: compartmentalization of corticostriatal input and striatonigral output systems. Nature 1984;311:461–4. 6207434 10.1038/311461a0

[56] Herkenham M, Pert CB. Mosaic distribution of opiate receptors, parafascicular projections and acetylcholinesterase in rat striatum. Nature 1981;291:415–8. 6165892 10.1038/291415a0

[57] Jimenez-Castellanos J, Graybiel AM. Subdivisions of the dopamine-containing A8-A9-A10 complex identified by their differential mesostriatal innervation of striosomes and extrastriosomal matrix. Neuroscience 1987;23:223–42. 3683862 10.1016/0306-4522(87)90285-5

[58] Gerfen CR. The neostriatal mosaic: striatal patch-matrix organization is related to cortical lamination. Science 1989;246:385–8. 2799392 10.1126/science.2799392

[59] Eblen F, Graybiel AM. Highly restricted origin of prefrontal cortical inputs to striosomes in the macaque monkey. J Neurosci 1995;15:5999–6013. 7666184 10.1523/JNEUROSCI.15-09-05999.1995 PMC6577677

[60] Kincaid AE, Wilson CJ. Corticostriatal innervation of the patch and matrix in the rat neostriatum. J Comp Neurol 1996;374:578–92. 8910736 10.1002/(SICI)1096-9861(19961028)374:4<578::AID-CNE7>3.0.CO;2-Z

[61] Crittenden JR, Graybiel AM. Basal Ganglia disorders associated with imbalances in the striatal striosome and matrix compartments. Front Neuroanat 2011;5:59. 21941467 10.3389/fnana.2011.00059 PMC3171104

[62] Langer LF, Graybiel AM. Distinct nigrostriatal projection systems innervate striosomes and matrix in the primate striatum. Brain Res 1989;498:344–50. 2477114 10.1016/0006-8993(89)91114-1

[63] Gerfen CR, Baimbridge KG, Thibault J. The neostriatal mosaic: III. Biochemical and developmental dissociation of patch-matrix mesostriatal systems. J Neurosci 1987;7:3935–44. 2891800 10.1523/JNEUROSCI.07-12-03935.1987 PMC6569111

[64] Olson L, Seiger A, Fuxe K. Heterogeneity of striatal and limbic dopamine innervation: highly fluorescent islands in developing and adult rats. Brain Res 1972;44:283–8. 4403485 10.1016/0006-8993(72)90385-x

[65] Tennyson VM, Barrett RE, Cohen G, Cote L, Heikkila R, Mytilineou C. The developing neostriatum of the rabbit: correlation of fluorescence histochemistry, electron microscopy, endogenous dopamine levels, and (3 H)dopamine uptake. Brain Res 1972;46:251–85. 4635366 10.1016/0006-8993(72)90019-4

[66] Poulin JF, Caronia G, Hofer C, Mapping projections of molecularly defined dopamine neuron subtypes using intersectional genetic approaches. Nat Neurosci 2018;21:1260–71. 30104732 10.1038/s41593-018-0203-4 PMC6342021

[67] Menegas W, Babayan BM, Uchida N, Watabe-Uchida M. Opposite initialization to novel cues in dopamine signaling in ventral and posterior striatum in mice. Elife 2017;6:e21886. 28054919 10.7554/eLife.21886 PMC5271609

[68] Williams SM, Goldman-Rakic P. Widespread origin of the primate mesofrontal dopamine system. Cerebral cortex 1998;8:321–45. 9651129 10.1093/cercor/8.4.321

[69] Lewis D, Sesack S. Chapter VI Dopamine systems in the primate brain. The Primate Nervous System, Part I. Elsevier; 1997. p. 263–375.

[70] Fallon JH, Loughlin SE. Substantia nigra. Rat Nervous System 1995.

[71] Watabe-Uchida M, Zhu L, Ogawa SK, Vamanrao A, Uchida N. Whole-brain mapping of direct inputs to midbrain dopamine neurons. Neuron 2012;74:858–73. 22681690 10.1016/j.neuron.2012.03.017

[72] Schultz W Getting formal with dopamine and reward. Neuron 2002;36:241–63. 12383780 10.1016/s0896-6273(02)00967-4

[73] Wittmann BC, Schott BH, Guderian S, Frey JU, Heinze HJ, Duzel E. Reward-related FMRI activation of dopaminergic midbrain is associated with enhanced hippocampus-dependent long-term memory formation. Neuron 2005;45:459–67. 15694331 10.1016/j.neuron.2005.01.010

[74] Matsumoto M, Hikosaka O. Two types of dopamine neuron distinctly convey positive and negative motivational signals. Nature 2009;459:837–41. 19448610 10.1038/nature08028 PMC2739096

[75] Bromberg-Martin ES, Matsumoto M, Hikosaka O. Dopamine in motivational control: rewarding, aversive, and alerting. Neuron 2010;68:815–34. 21144997 10.1016/j.neuron.2010.11.022 PMC3032992

[76] Redgrave P, Prescott TJ, Gurney K. The basal ganglia: a vertebrate solution to the selection problem? Neuroscience 1999;89:1009–23. 10362291 10.1016/s0306-4522(98)00319-4

[77] Horvitz JC. Mesolimbocortical and nigrostriatal dopamine responses to salient non-reward events. Neuroscience 2000;96:651–6. 10727783 10.1016/s0306-4522(00)00019-1

[78] Chiara G Nucleus accumbens shell and core dopamine: differential role in behavior and addiction. Behav Brain Res 2002;137:75–114. 12445717 10.1016/s0166-4328(02)00286-3

[79] Pezze MA, Feldon J. Mesolimbic dopaminergic pathways in fear conditioning. Prog Neurobiol 2004;74:301–20. 15582224 10.1016/j.pneurobio.2004.09.004

[80] Lisman JE, Grace AA. The hippocampal-VTA loop: controlling the entry of information into long-term memory. Neuron 2005;46:703–13. 15924857 10.1016/j.neuron.2005.05.002

[81] Redgrave P, Gurney K. The short-latency dopamine signal: a role in discovering novel actions? Nat Rev Neurosci 2006;7:967–75. 17115078 10.1038/nrn2022

[82] Chinta SJ, Andersen JK. Dopaminergic neurons. Int J Biochem Cell Biol 2005;37:942–6. 15743669 10.1016/j.biocel.2004.09.009

[83] Iversen SD, Iversen LL. Dopamine: 50 years in perspective. Trends Neurosci 2007;30:188–93. 17368565 10.1016/j.tins.2007.03.002

[84] Matsumoto N, Hanakawa T, Maki S, Graybiel AM, Kimura M. Role of [corrected] nigrostriatal dopamine system in learning to perform sequential motor tasks in a predictive manner. J Neurophysiol 1999;82:978–98. 10444692 10.1152/jn.1999.82.2.978

[85] Gambhir H, Mathur R, Behari M. Progressive impairment in motor skill learning at 12 and 20 weeks post 6-OHDA-SNc lesion in rats. Parkinsonism Relat Disord 2011;17:476–8. 21367640 10.1016/j.parkreldis.2010.12.017

[86] Nomoto K, Schultz W, Watanabe T, Sakagami M. Temporally extended dopamine responses to perceptually demanding reward-predictive stimuli. J Neurosci 2010;30:10692–702. 20702700 10.1523/JNEUROSCI.4828-09.2010 PMC3297489

[87] Mirenowicz J, Schultz W. Preferential activation of midbrain dopamine neurons by appetitive rather than aversive stimuli. Nature 1996;379:449–51. 8559249 10.1038/379449a0

[88] Zhang Y, Larcher KM, Misic B, Dagher A. Anatomical and functional organization of the human substantia nigra and its connections. Elife 2017;6:e26653. 28826495 10.7554/eLife.26653 PMC5606848

[89] Dagher A, Robbins TW. Personality, addiction, dopamine: insights from Parkinson’s disease. Neuron 2009;61:502–10. 19249271 10.1016/j.neuron.2009.01.031

[90] Dalley JW, Robbins TW. Fractionating impulsivity: neuropsychiatric implications. Nat Rev Neurosci 2017;18:158–71. 10.1038/nrn.2017.8 28209979

[91] Morris LS, Kundu P, Dowell N, Fronto-striatal organization: Defining functional and microstructural substrates of behavioural flexibility. Cortex 2016;74:118–33. 26673945 10.1016/j.cortex.2015.11.004 PMC4729321

[92] Schiemann J, Schlaudraff F, Klose V, K-ATP channels in dopamine substantia nigra neurons control bursting and novelty-induced exploration. Nat Neurosci 2012;15:1272–80. 22902720 10.1038/nn.3185 PMC4242970

[93] Jin X, Costa RM. Start/stop signals emerge in nigrostriatal circuits during sequence learning. Nature 2010;466:457–62. 20651684 10.1038/nature09263 PMC3477867

[94] Schultz W Multiple dopamine functions at different time courses. Annu Rev Neurosci 2007;30:259–88. 17600522 10.1146/annurev.neuro.28.061604.135722

[95] Yin HH, Ostlund SB, Balleine BW. Reward-guided learning beyond dopamine in the nucleus accumbens: the integrative functions of cortico-basal ganglia networks. Eur J Neurosci 2008;28:1437–48. 18793321 10.1111/j.1460-9568.2008.06422.x PMC2756656

[96] Cohen MX, Frank MJ. Neurocomputational models of basal ganglia function in learning, memory and choice. Behav Brain Res 2009;199:141–56. 18950662 10.1016/j.bbr.2008.09.029 PMC2762323

[97] Han JS, McMahan RW, Holland P, Gallagher M. The role of an amygdalo-nigrostriatal pathway in associative learning. J Neurosci 1997;17:3913–9. 9133409 10.1523/JNEUROSCI.17-10-03913.1997 PMC6573694

[98] Lee HJ, Groshek F, Petrovich GD, Cantalini JP, Gallagher M, Holland PC. Role of amygdalo-nigral circuitry in conditioning of a visual stimulus paired with food. J Neurosci 2005;25:3881–8. 15829640 10.1523/JNEUROSCI.0416-05.2005 PMC1201483

[99] El-Amamy H, Holland PC. Dissociable effects of disconnecting amygdala central nucleus from the ventral tegmental area or substantia nigra on learned orienting and incentive motivation. Eur J Neurosci 2007;25:1557–67. 17425582 10.1111/j.1460-9568.2007.05402.x PMC2850064

[100] Hall J, Parkinson JA, Connor TM, Dickinson A, Everitt BJ. Involvement of the central nucleus of the amygdala and nucleus accumbens core in mediating Pavlovian influences on instrumental behaviour. Eur J Neurosci 2001;13:1984–92. 11403692 10.1046/j.0953-816x.2001.01577.x

[101] Corbit LH, Balleine BW. Double dissociation of basolateral and central amygdala lesions on the general and outcome-specific forms of pavlovian-instrumental transfer. J Neurosci 2005;25:962–70. 15673677 10.1523/JNEUROSCI.4507-04.2005 PMC6725628

[102] Ikemoto S Dopamine reward circuitry: two projection systems from the ventral midbrain to the nucleus accumbens-olfactory tubercle complex. Brain Res Rev 2007;56:27–78. 17574681 10.1016/j.brainresrev.2007.05.004 PMC2134972

[103] Roeper J. Dissecting the diversity of midbrain dopamine neurons. Trends Neurosci 2013;36:336–42. 23582338 10.1016/j.tins.2013.03.003

[104] Morales M, Margolis EB. Ventral tegmental area: cellular heterogeneity, connectivity and behaviour. Nat Rev Neurosci 2017;18:73–85. 28053327 10.1038/nrn.2016.165

[105] Lammel S, Ion Daniela I, Roeper J, Malenka Robert C. Projection-specific modulation of dopamine neuron synapses by aversive and rewarding stimuli. Neuron 2011;70:855–62. 21658580 10.1016/j.neuron.2011.03.025 PMC3112473

[106] Carmichael K, Evans RC, Lopez E, Function and regulation of ALDH1A1-positive nigrostriatal dopaminergic neurons in motor control and Parkinson’s disease. Front Neural Circuits 2021;15:644776. 34079441 10.3389/fncir.2021.644776 PMC8165242

[107] Marchitti SA, Deitrich RA, Vasiliou V. Neurotoxicity and metabolism of the catecholamine-derived 3,4-dihydroxyphenylacetaldehyde and 3,4-dihydroxyphenylglycolaldehyde: the role of aldehyde dehydrogenase. Pharmacol Rev 2007;59:125–50. 17379813 10.1124/pr.59.2.1

[108] Fearnley JM, Lees AJ. Ageing and Parkinson’s disease: substantia nigra regional selectivity. Brain 1991;114 (Pt 5):2283–301. 1933245 10.1093/brain/114.5.2283

[109] Sgobio C, Wu J, Zheng W, Aldehyde dehydrogenase 1-positive nigrostriatal dopaminergic fibers exhibit distinct projection pattern and dopamine release dynamics at mouse dorsal striatum. Sci Rep 2017;7:5283. 28706191 10.1038/s41598-017-05598-1 PMC5509666

[110] Pan J, Yu J, Sun L, ALDH1A1 regulates postsynaptic mu-opioid receptor expression in dorsal striatal projection neurons and mitigates dyskinesia through transsynaptic retinoic acid signaling. Sci Rep 2019;9:3602. 30837649 10.1038/s41598-019-40326-x PMC6401150

[111] Evans RC, Twedell EL, Zhu M, Ascencio J, Zhang R, Khaliq ZM. Functional dissection of basal ganglia inhibitory inputs onto substantia nigra dopaminergic neurons. Cell Rep 2020;32:108156. 32937133 10.1016/j.celrep.2020.108156 PMC9887718

[112] Graves SM, Xie Z, Stout KA, Dopamine metabolism by a monoamine oxidase mitochondrial shuttle activates the electron transport chain. Nat Neurosci 2020;23:15–20. 31844313 10.1038/s41593-019-0556-3 PMC7257994

[113] Nair-Roberts RG, Chatelain-Badie SD, Benson E, White-Cooper H, Bolam JP, Ungless MA. Stereological estimates of dopaminergic, GABAergic and glutamatergic neurons in the ventral tegmental area, substantia nigra and retrorubral field in the rat. Neuroscience 2008;152:1024–31. 18355970 10.1016/j.neuroscience.2008.01.046 PMC2575227

[114] Taylor SR, Badurek S, Dileone RJ, Nashmi R, Minichiello L, Picciotto MR. GABAergic and glutamatergic efferents of the mouse ventral tegmental area. J Comp Neurol 2014;522:3308–34. 24715505 10.1002/cne.23603 PMC4107038

[115] Breton JM, Charbit AR, Snyder BJ, Relative contributions and mapping of ventral tegmental area dopamine and GABA neurons by projection target in the rat. J Comp Neurol 2019;527:916–41. 30393861 10.1002/cne.24572 PMC6347508

[116] Fu Y, Paxinos G, Watson C, Halliday GM. The substantia nigra and ventral tegmental dopaminergic neurons from development to degeneration. J Chem Neuroanat 2016;76:98–107. 26859066 10.1016/j.jchemneu.2016.02.001

[117] Halliday GM, Leverenz JB, Schneider JS, Adler CH. The neurobiological basis of cognitive impairment in Parkinson’s disease. Mov Disord 2014;29:634–50. 24757112 10.1002/mds.25857 PMC4049032

[118] Watson GS, Leverenz JB. Profile of cognitive impairment in Parkinson’s disease. Brain Pathol 2010;20:640–5. 20522089 10.1111/j.1750-3639.2010.00373.x PMC3049172

[119] Lanciego JL, Luquin N, Obeso JA. Functional neuroanatomy of the basal ganglia. Cold Spring Harb Perspect Med 2012;2:a009621. 23071379 10.1101/cshperspect.a009621 PMC3543080

[120] Settell ML, Testini P, Cho S, Functional circuitry effect of ventral tegmental area deep brain stimulation: imaging and neurochemical evidence of mesocortical and mesolimbic pathway modulation. Front Neurosci 2017;11:104. 28316564 10.3389/fnins.2017.00104 PMC5334355

[121] Ballard IC, Murty VP, Carter RM, MacInnes JJ, Huettel SA, Adcock RA. Dorsolateral prefrontal cortex drives mesolimbic dopaminergic regions to initiate motivated behavior. J Neurosci 2011;31:10340–6. 21753011 10.1523/JNEUROSCI.0895-11.2011 PMC3182466

[122] Hauser TU, Eldar E, Dolan RJ. Separate mesocortical and mesolimbic pathways encode effort and reward learning signals. Proc Natl Acad Sci U S A 2017;114:E7395–404. 28808037 10.1073/pnas.1705643114 PMC5584432

[123] Halbout B, Marshall AT, Azimi A, Mesolimbic dopamine projections mediate cue-motivated reward seeking but not reward retrieval in rats. Elife 2019;8:e43551. 31107241 10.7554/eLife.43551 PMC6548499

[124] Mingote S, Amsellem A, Kempf A, Rayport S, Chuhma N. Dopamine-glutamate neuron projections to the nucleus accumbens medial shell and behavioral switching. Neurochem Int 2019;129:104482. 31170424 10.1016/j.neuint.2019.104482 PMC6855309

[125] Wanat MJ, Willuhn I, Clark JJ, Phillips PE. Phasic dopamine release in appetitive behaviors and drug addiction. Curr Drug Abuse Rev 2009;2:195–213. 19630749 10.2174/1874473710902020195 PMC2877500

[126] Marinelli M, McCutcheon JE. Heterogeneity of dopamine neuron activity across traits and states. Neuroscience 2014;282:176–97. 25084048 10.1016/j.neuroscience.2014.07.034 PMC4312268

[127] Zhou Y, Bunney BS, Shi WX. Differential effects of cocaine on firing rate and pattern of dopamine neurons: role of alpha1 receptors and comparison with L-dopa and apomorphine. J Pharmacol Exp Ther 2006;317:196–201. 16330495 10.1124/jpet.105.094045

[128] Fu Y, Yuan Y, Halliday G, Rusznak Z, Watson C, Paxinos G. A cytoarchitectonic and chemoarchitectonic analysis of the dopamine cell groups in the substantia nigra, ventral tegmental area, and retrorubral field in the mouse. Brain Struct Funct 2012;217:591–612. 21935672 10.1007/s00429-011-0349-2

[129] Anderegg A, Poulin JF, Awatramani R. Molecular heterogeneity of midbrain dopaminergic neurons--moving toward single cell resolution. FEBS Lett 2015;589:3714–26. 26505674 10.1016/j.febslet.2015.10.022 PMC4679573

[130] Cai LX, Pizano K, Gundersen GW, Distinct signals in medial and lateral VTA dopamine neurons modulate fear extinction at different times. Elife 2020;9:e54936. 32519951 10.7554/eLife.54936 PMC7363446

[131] Beier KT, Steinberg EE, DeLoach KE, Circuit architecture of VTA dopamine neurons revealed by systematic input-output mapping. Cell 2015;162:622–34. 26232228 10.1016/j.cell.2015.07.015 PMC4522312

[132] Del-Fava F, Hasue RH, Ferreira JG, Shammah-Lagnado SJ. Efferent connections of the rostral linear nucleus of the ventral tegmental area in the rat. Neuroscience 2007;145:1059–76. 17270353 10.1016/j.neuroscience.2006.12.039

[133] Vaaga CE, Borisovska M, Westbrook GL. Dual-transmitter neurons: functional implications of co-release and co-transmission. Curr Opin Neurobiol 2014;29:25–32. 24816154 10.1016/j.conb.2014.04.010 PMC4231002

[134] Lammel S, Lim BK, Ran C, Input-specific control of reward and aversion in the ventral tegmental area. Nature 2012;491:212–7. 23064228 10.1038/nature11527 PMC3493743

[135] Lammel S, Lim BK, Malenka RC. Reward and aversion in a heterogeneous midbrain dopamine system. Neuropharmacology 2014;76 Pt B:351–9. 23578393 10.1016/j.neuropharm.2013.03.019 PMC3778102

[136] Alberico SL, Cassell MD, Narayanan NS. The vulnerable ventral tegmental area in Parkinson’s disease. Basal Ganglia 2015;5:51–5. 26251824 10.1016/j.baga.2015.06.001 PMC4523275

[137] Fahn S Description of Parkinson’s disease as a clinical syndrome. Ann N Y Acad Sci 2003;991:1–14. 10.1111/j.1749-6632.2003.tb07458.x 12846969

[138] Zampese E, Galtieri D, Schumacker P, Surmeier D. Determinants of Selective Vulnerability of Dopamine Neurons in Parkinson's Disease. Handbook of Basal Ganglia Structure and Function, Second Edition. Elsevier; 2016. p. 821–37.

[139] Bisaglia M, Filograna R, Beltramini M, Bubacco L. Are dopamine derivatives implicated in the pathogenesis of Parkinson’s disease? Ageing Res Rev 2014;13:107–14. 24389159 10.1016/j.arr.2013.12.009

[140] Fahn S, Jankovic J, Hallett M. Current concepts on the etiology and pathogenesis of Parkinson disease. Principles and Practice of Movement Disorders. Elsevier; 2011. p. 93–118.

[141] Alter SP, Lenzi GM, Bernstein AI, Miller GW. Vesicular integrity in Parkinson’s disease. Curr Neurol Neurosci Rep 2013;13:362. 23690026 10.1007/s11910-013-0362-3 PMC4019229

[142] Sanders LH, Timothy Greenamyre J. Oxidative damage to macromolecules in human Parkinson disease and the rotenone model. Free Radic Biol Med 2013;62:111–20. 23328732 10.1016/j.freeradbiomed.2013.01.003 PMC3677955

[143] Asanuma M, Miyazaki I, Diaz-Corrales FJ, Ogawa N. Quinone formation as dopaminergic neuron-specific oxidative stress in the pathogenesis of sporadic Parkinson’s disease and neurotoxin-induced parkinsonism. Acta Med Okayama 2004;58:221–33. 15666991 10.18926/AMO/32105

[144] Wang N, Wang Y, Yu G, Yuan C, Ma J. Quinoprotein adducts accumulate in the substantia nigra of aged rats and correlate with dopamine-induced toxicity in SH-SY5Y cells. Neurochem Res 2011;36:2169–75. 21785836 10.1007/s11064-011-0541-z

[145] Segura-Aguilar J, Paris I, Munoz P, Ferrari E, Zecca L, Zucca FA. Protective and toxic roles of dopamine in Parkinson’s disease. J Neurochem 2014;129:898–915. 24548101 10.1111/jnc.12686

[146] Zucca FA, Basso E, Cupaioli FA, Neuromelanin of the human substantia nigra: an update. Neurotox Res 2014;25:13–23. 24155156 10.1007/s12640-013-9435-y

[147] Sulzer D, Bogulavsky J, Larsen KE, Neuromelanin biosynthesis is driven by excess cytosolic catecholamines not accumulated by synaptic vesicles. Proc Natl Acad Sci U S A 2000;97:11869–74. 11050221 10.1073/pnas.97.22.11869 PMC17261

[148] Sulzer D, Zecca L. Intraneuronal dopamine-quinone synthesis: a review. Neurotox Res 2000;1:181–95. 12835101 10.1007/BF03033289

[149] Zecca L, Zucca FA, Wilms H, Sulzer D. Neuromelanin of the substantia nigra: a neuronal black hole with protective and toxic characteristics. Trends Neurosci 2003;26:578–80. 14585596 10.1016/j.tins.2003.08.009

[150] Mann DM, Yates PO. Possible role of neuromelanin in the pathogenesis of Parkinson’s disease. Mech Ageing Dev 1983;21:193–203. 6865505 10.1016/0047-6374(83)90074-x

[151] Mosharov EV, Staal RG, Bove J, Alpha-synuclein overexpression increases cytosolic catecholamine concentration. J Neurosci 2006;26:9304–11. 16957086 10.1523/JNEUROSCI.0519-06.2006 PMC6674515

[152] Conway KA, Rochet JC, Bieganski RM, Lansbury PT Jr. Kinetic stabilization of the alpha-synuclein protofibril by a dopamine-alpha-synuclein adduct. Science 2001;294:1346–9. 11701929 10.1126/science.1063522

[153] Rochet JC, Outeiro TF, Conway KA, Interactions among alpha-synuclein, dopamine, and biomembranes: some clues for understanding neurodegeneration in Parkinson’s disease. J Mol Neurosci 2004;23:23–34. 15126689 10.1385/jmn:23:1-2:023

[154] Sulzer D Clues to how alpha-synuclein damages neurons in Parkinson’s disease. Mov Disord 2010;25 Suppl 1:S27–31. 20187229 10.1002/mds.22639

[155] Martinez-Vicente M, Talloczy Z, Kaushik S, Dopamine-modified alpha-synuclein blocks chaperone-mediated autophagy. J Clin Invest 2008;118:777–88. 18172548 10.1172/JCI32806 PMC2157565

[156] Burke WJ, Kumar VB, Pandey N, Aggregation of alpha-synuclein by DOPAL, the monoamine oxidase metabolite of dopamine. Acta neuropathologica 2008;115:193–203. 17965867 10.1007/s00401-007-0303-9

[157] Fahn S, Cohen G. The oxidant stress hypothesis in Parkinson’s disease: evidence supporting it. Ann Neurol 1992;32:804–12. 1471873 10.1002/ana.410320616

[158] Kordower JH, Olanow CW, Dodiya HB, Disease duration and the integrity of the nigrostriatal system in Parkinson’s disease. Brain 2013;136:2419–31. 23884810 10.1093/brain/awt192 PMC3722357

[159] Koppaka V, Thompson DC, Chen Y, Aldehyde dehydrogenase inhibitors: a comprehensive review of the pharmacology, mechanism of action, substrate specificity, and clinical application. Pharmacol Rev 2012;64:520–39. 22544865 10.1124/pr.111.005538 PMC3400832

[160] Burke RE. Intracellular signalling pathways in dopamine cell death and axonal degeneration. Prog Brain Res 2010;183:79–97. 20696316 10.1016/S0079-6123(10)83005-5 PMC3088517

[161] Goldstein DS, Kopin IJ, Sharabi Y. Catecholamine autotoxicity. Implications for pharmacology and therapeutics of Parkinson disease and related disorders. Pharmacol Ther 2014;144:268–82. 24945828 10.1016/j.pharmthera.2014.06.006 PMC4591072

[162] Grunblatt E, Ruder J, Monoranu CM, Riederer P, Youdim MB, Mandel SA. Differential alterations in metabolism and proteolysis-related proteins in human Parkinson’s disease substantia nigra. Neurotox Res 2017;33:560–8. 29218503 10.1007/s12640-017-9843-5

[163] Wey MC, Fernandez E, Martinez PA, Sullivan P, Goldstein DS, Strong R. Neurodegeneration and motor dysfunction in mice lacking cytosolic and mitochondrial aldehyde dehydrogenases: implications for Parkinson’s disease. PloS one 2012;7:e31522. 22384032 10.1371/journal.pone.0031522 PMC3284575

[164] Fan HH, Guo Q, Zheng J, ALDH1A1 genetic variations may modulate risk of Parkinson’s disease in Han Chinese Population. Front Neurosci 2021;15:620929. 33815038 10.3389/fnins.2021.620929 PMC8017280

[165] Fitzmaurice AG, Rhodes SL, Lulla A, Aldehyde dehydrogenase inhibition as a pathogenic mechanism in Parkinson disease. Proc Natl Acad Sci U S A 2013;110:636–41. 23267077 10.1073/pnas.1220399110 PMC3545765

[166] Burke RE, O’Malley K. Axon degeneration in Parkinson’s disease. Exp Neurol 2013;246:72–83. 22285449 10.1016/j.expneurol.2012.01.011 PMC3340476

[167] Garcia-Reitbock P, Anichtchik O, Bellucci A, SNARE protein redistribution and synaptic failure in a transgenic mouse model of Parkinson’s disease. Brain 2010;133:2032–44. 20534649 10.1093/brain/awq132 PMC2892942

[168] Kish SJ, Shannak K, Hornykiewicz O. Uneven pattern of dopamine loss in the striatum of patients with idiopathic Parkinson’s disease. Pathophysiologic and clinical implications. N Engl J Med 1988;318:876–80. 3352672 10.1056/NEJM198804073181402

[169] Volpicelli-Daley LA, Luk KC, Patel TP, Exogenous alpha-synuclein fibrils induce Lewy body pathology leading to synaptic dysfunction and neuron death. Neuron 2011;72:57–71. 21982369 10.1016/j.neuron.2011.08.033 PMC3204802

[170] Arbuthnott GW, Wickens J. Space, time and dopamine. Trends Neurosci 2007;30:62–9. 17173981 10.1016/j.tins.2006.12.003

[171] Groves PM, Linder JC, Young SJ. 5-hydroxydopamine-labeled dopaminergic axons: three-dimensional reconstructions of axons, synapses and postsynaptic targets in rat neostriatum. Neuroscience 1994;58:593–604. 8170539 10.1016/0306-4522(94)90084-1

[172] Matsuda W, Furuta T, Nakamura KC, Single nigrostriatal dopaminergic neurons form widely spread and highly dense axonal arborizations in the neostriatum. J Neurosci 2009;29:444–53. 19144844 10.1523/JNEUROSCI.4029-08.2009 PMC6664950

[173] Prensa L, Parent A. The nigrostriatal pathway in the rat: A single-axon study of the relationship between dorsal and ventral tier nigral neurons and the striosome/matrix striatal compartments. J Neurosci 2001;21:7247–60. 11549735 10.1523/JNEUROSCI.21-18-07247.2001 PMC6762986

[174] Bolam JP, Pissadaki EK. Living on the edge with too many mouths to feed: why dopamine neurons die. Mov Disord 2012;27:1478–83. 23008164 10.1002/mds.25135 PMC3504389

[175] Pissadaki EK, Bolam JP. The energy cost of action potential propagation in dopamine neurons: clues to susceptibility in Parkinson’s disease. Front Comput Neurosci 2013;7:13. 23515615 10.3389/fncom.2013.00013 PMC3600574

[176] Ashrafi G, Schlehe JS, LaVoie MJ, Schwarz TL. Mitophagy of damaged mitochondria occurs locally in distal neuronal axons and requires PINK1 and Parkin. J Cell Biol 2014;206:655–70. 25154397 10.1083/jcb.201401070 PMC4151150

[177] Vos KJ, Grierson AJ, Ackerley S, Miller CC. Role of axonal transport in neurodegenerative diseases. Annu Rev Neurosci 2008;31:151–73. 18558852 10.1146/annurev.neuro.31.061307.090711

[178] Millecamps S, Julien JP. Axonal transport deficits and neurodegenerative diseases. Nat Rev Neurosci 2013;14:161–76. 23361386 10.1038/nrn3380

[179] Morfini GA, Burns M, Binder LI, Axonal transport defects in neurodegenerative diseases. J Neurosci 2009;29:12776–86. 19828789 10.1523/JNEUROSCI.3463-09.2009 PMC2801051

[180] Salinas S, Bilsland LG, Schiavo G. Molecular landmarks along the axonal route: axonal transport in health and disease. Curr Opin Cell Biol 2008;20:445–53. 18495455 10.1016/j.ceb.2008.04.002

[181] Surmeier DJ, Ding J, Day M, Wang Z, Shen W. D1 and D2 dopamine-receptor modulation of striatal glutamatergic signaling in striatal medium spiny neurons. Trends Neurosci 2007;30:228–35. 17408758 10.1016/j.tins.2007.03.008

[182] Chan CS, Guzman JN, Ilijic E, ‘Rejuvenation’ protects neurons in mouse models of Parkinson’s disease. Nature 2007;447:1081–6. 17558391 10.1038/nature05865

[183] Grace AA, Bunney BS. Intracellular and extracellular electrophysiology of nigral dopaminergic neurons--3. Evidence for electrotonic coupling. Neuroscience 1983;10:333–48. 6633865 10.1016/0306-4522(83)90137-9

[184] Guzman JN, Sanchez-Padilla J, Chan CS, Surmeier DJ. Robust pacemaking in substantia nigra dopaminergic neurons. J Neurosci 2009;29:11011–9. 19726659 10.1523/JNEUROSCI.2519-09.2009 PMC2784968

[185] Neuhoff H, Neu A, Liss B, Roeper J. I(h) channels contribute to the different functional properties of identified dopaminergic subpopulations in the midbrain. J Neurosci 2002;22:1290–302. 11850457 10.1523/JNEUROSCI.22-04-01290.2002 PMC6757558

[186] Puopolo M, Raviola E, Bean BP. Roles of subthreshold calcium current and sodium current in spontaneous firing of mouse midbrain dopamine neurons. J Neurosci 2007;27:645–56. 17234596 10.1523/JNEUROSCI.4341-06.2007 PMC6672803

[187] Wilson CJ, Callaway JC. Coupled oscillator model of the dopaminergic neuron of the substantia nigra. J Neurophysiol 2000;83:3084–100. 10805703 10.1152/jn.2000.83.5.3084

[188] Grace AA, Bunney BS. The control of firing pattern in nigral dopamine neurons: single spike firing. J Neurosci 1984;4:2866–76. 6150070 10.1523/JNEUROSCI.04-11-02866.1984 PMC6564731

[189] Overton PG, Clark D. Burst firing in midbrain dopaminergic neurons. Brain Res Brain Res Rev 1997;25:312–34. 9495561 10.1016/s0165-0173(97)00039-8

[190] Grace AA, Bunney BS. The control of firing pattern in nigral dopamine neurons: burst firing. J Neurosci 1984;4:2877–90. 6150071 10.1523/JNEUROSCI.04-11-02877.1984 PMC6564720

[191] Baughman JM, Perocchi F, Girgis HS, Integrative genomics identifies MCU as an essential component of the mitochondrial calcium uniporter. Nature 2011;476:341–5. 21685886 10.1038/nature10234 PMC3486726

[192] Stefani D, Raffaello A, Teardo E, Szabo I, Rizzuto R. A forty-kilodalton protein of the inner membrane is the mitochondrial calcium uniporter. Nature 2011;476:336–40. 21685888 10.1038/nature10230 PMC4141877

[193] Griffiths EJ, Rutter GA. Mitochondrial calcium as a key regulator of mitochondrial ATP production in mammalian cells. Biochim Biophys Acta 2009;1787:1324–33. 19366607 10.1016/j.bbabio.2009.01.019

[194] McCormack JG, Denton RM. Mitochondrial Ca2+ transport and the role of intramitochondrial Ca2+ in the regulation of energy metabolism. Dev Neurosci 1993;15:165–73. 7805568 10.1159/000111332

[195] McCormack JG, Halestrap AP, Denton RM. Role of calcium ions in regulation of mammalian intramitochondrial metabolism. Physiol Rev 1990;70:391–425. 2157230 10.1152/physrev.1990.70.2.391

[196] Scheibye-Knudsen M, Mitchell SJ, Fang EF, A high-fat diet and NAD(+) activate Sirt1 to rescue premature aging in cockayne syndrome. Cell Metab 2014;20:840–55. 25440059 10.1016/j.cmet.2014.10.005 PMC4261735

[197] Nicholls DG, Budd SL. Mitochondria and neuronal survival. Physiol Rev 2000;80:315–60. 10617771 10.1152/physrev.2000.80.1.315

[198] Lin MT, Beal MF. Mitochondrial dysfunction and oxidative stress in neurodegenerative diseases. Nature 2006;443:787–95. 17051205 10.1038/nature05292

[199] Reeve AK, Krishnan KJ, Turnbull D. Mitochondrial DNA mutations in disease, aging, and neurodegeneration. Ann N Y Acad Sci 2008;1147:21–9. 19076427 10.1196/annals.1427.016

[200] Follett J, Norwood SJ, Hamilton NA, The Vps35 D620N mutation linked to Parkinson’s disease disrupts the cargo sorting function of retromer. Traffic 2014;15:230–44. 24152121 10.1111/tra.12136

[201] Nielsen MS, Vorum H, Lindersson E, Jensen PH. Ca2+ binding to alpha-synuclein regulates ligand binding and oligomerization. J Biol Chem 2001;276:22680–4. 11312271 10.1074/jbc.M101181200

[202] Rcom-H’cheo-Gauthier A, Goodwin J, Pountney DL: Interactions between calcium and alpha-synuclein in neurodegeneration. Biomolecules 2014;4:795–811. 25256602 10.3390/biom4030795 PMC4192672

[203] Foehring RC, Zhang XF, Lee JC, Callaway JC. Endogenous calcium buffering capacity of substantia nigral dopamine neurons. J Neurophysiol 2009;102:2326–33. 19675297 10.1152/jn.00038.2009 PMC2775382

[204] Subramaniam M, Althof D, Gispert S, Mutant alpha-synuclein enhances firing frequencies in dopamine substantia nigra neurons by oxidative impairment of A-type potassium channels. J Neurosci 2014;34:13586–99. 25297088 10.1523/JNEUROSCI.5069-13.2014 PMC6608377

[205] Monyer H, Burnashev N, Laurie DJ, Sakmann B, Seeburg PH. Developmental and regional expression in the rat brain and functional properties of four NMDA receptors. Neuron 1994;12:529–40. 7512349 10.1016/0896-6273(94)90210-0

[206] Traynelis SF, Wollmuth LP, McBain CJ, Glutamate receptor ion channels: structure, regulation, and function. Pharmacol Rev 2010;62:405–96. 20716669 10.1124/pr.109.002451 PMC2964903

[207] Standaert DG, Testa CM, Penney JB Jr, Young AB. Alternatively spliced isoforms of the NMDAR1 glutamate receptor subunit: differential expression in the basal ganglia of the rat. Neurosci Lett 1993;152:161–4. 8515871 10.1016/0304-3940(93)90508-i

[208] Morikawa H, Khodakhah K, Williams JT. Two intracellular pathways mediate metabotropic glutamate receptor-induced Ca2+ mobilization in dopamine neurons. J Neurosci 2003;23:149–57. 12514211 10.1523/JNEUROSCI.23-01-00149.2003 PMC1408315

[209] Lawson LJ, Perry VH, Dri P, Gordon S. Heterogeneity in the distribution and morphology of microglia in the normal adult mouse brain. Neuroscience 1990;39:151–70. 2089275 10.1016/0306-4522(90)90229-w

[210] Zecca L, Casella L, Albertini A, Neuromelanin can protect against iron-mediated oxidative damage in system modeling iron overload of brain aging and Parkinson’s disease. J Neurochem 2008;106:1866–75. 18624918 10.1111/j.1471-4159.2008.05541.x

[211] Zhang W, Phillips K, Wielgus AR, Neuromelanin activates microglia and induces degeneration of dopaminergic neurons: implications for progression of Parkinson’s disease. Neurotox Res 2011;19:63–72. 19957214 10.1007/s12640-009-9140-z PMC3603276

[212] McGeer PL, McGeer EG. Glial reactions in Parkinson’s disease. Mov Disord 2008;23:474–83. 18044695 10.1002/mds.21751

[213] Phani S, Re DB, Przedborski S. The role of the innate immune system in ALS. Front Pharmacol 2012;3:150. 22912616 10.3389/fphar.2012.00150 PMC3418523

[214] Narayanan NS, Rodnitzky RL, Uc EY. Prefrontal dopamine signaling and cognitive symptoms of Parkinson’s disease. Rev Neurosci 2013;24:267–78. 23729617 10.1515/revneuro-2013-0004 PMC3836593

[215] Winter C, von Rumohr A, Mundt A, Lesions of dopaminergic neurons in the substantia nigra pars compacta and in the ventral tegmental area enhance depressive-like behavior in rats. Behav Brain Res 2007;184:133–41. 17698212 10.1016/j.bbr.2007.07.002

[216] Faivre F, Sanchez-Catalan MJ, Dovero S, Ablation of the tail of the ventral tegmental area compensates symptoms in an experimental model of Parkinson’s disease. Neurobiol Dis 2020;139:104818. 32087289 10.1016/j.nbd.2020.104818

[217] Guo L, Xiong H, Kim JI, Dynamic rewiring of neural circuits in the motor cortex in mouse models of Parkinson’s disease. Nat Neurosci 2015;18:1299–309. 26237365 10.1038/nn.4082 PMC4551606

[218] Petrou M, Kotagal V, Bohnen NI. An update on brain imaging in parkinsonian dementia. Imaging Med 2012;4:201–13. 22768021 10.2217/iim.12.10 PMC3387991

[219] Goldman JG, Stebbins GT, Bernard B, Stoub TR, Goetz CG, deToledo-Morrell L. Entorhinal cortex atrophy differentiates Parkinson’s disease patients with and without dementia. Mov Disord 2012;27:727–34. 22410753 10.1002/mds.24938 PMC3366041

[220] McNamara CG, Dupret D. Two sources of dopamine for the hippocampus. Trends Neurosci 2017;40:383–4. 28511793 10.1016/j.tins.2017.05.005 PMC5489110

[221] Narayanan NS, Land BB, Solder JE, Deisseroth K, DiLeone RJ. Prefrontal D1 dopamine signaling is required for temporal control. Proc Natl Acad Sci U S A 2012;109:20726–31. 23185016 10.1073/pnas.1211258109 PMC3528521

[222] Parker KL, Alberico SL, Miller AD, Narayanan NS. Prefrontal D1 dopamine signaling is necessary for temporal expectation during reaction time performance. Neuroscience 2013;255:246–54. 24120554 10.1016/j.neuroscience.2013.09.057 PMC3856920

[223] Parkinson GM, Dayas CV, Smith DW. Increased mitochondrial DNA deletions in substantia nigra dopamine neurons of the aged rat. Curr Aging Sci 2014;7:155–60. 25612740 10.2174/1874609808666150122150850

[224] Backman L, Farde L. Dopamine and cognitive functioning: brain imaging findings in Huntington’s disease and normal aging. Scand J Psychol 2001;42:287–96. 11501742 10.1111/1467-9450.00238

[225] Kaasinen V, Rinne JO. Functional imaging studies of dopamine system and cognition in normal aging and Parkinson’s disease. Neurosci Biobehav Rev 2002;26:785–93. 12470690 10.1016/s0149-7634(02)00065-9

[226] Leranth C, Roth RH, Elswoth JD, Naftolin F, Horvath TL, Redmond DE. Estrogen is essential for maintaining nigrostriatal dopamine neurons in primates: Implications for Parkinson’s disease and memory. J Neurosci 2000;20:8604–09. 11102464 10.1523/JNEUROSCI.20-23-08604.2000 PMC6773080

[227] Shohamy D, Wimmer GE. Dopamine and the cost of aging. Nat Neurosci 2013;16:519–21. 23619753 10.1038/nn.3385

[228] Halliday GM, McCann H. The progression of pathology in Parkinson’s disease. Ann N Y Acad Sci 2010;1184:188–95. 20146698 10.1111/j.1749-6632.2009.05118.x

[229] Kanaan NM, Kordower JH, Collier TJ. Age-related changes in dopamine transporters and accumulation of 3-nitrotyrosine in rhesus monkey midbrain dopamine neurons: relevance in selective neuronal vulnerability to degeneration. Eur J Neurosci 2008;27:3205–15. 18598263 10.1111/j.1460-9568.2008.06307.x PMC3391583

[230] Sulzer D, Cragg SJ, Rice ME. Striatal dopamine neurotransmission: regulation of release and uptake. Basal Ganglia 2016;6:123–48. 27141430 10.1016/j.baga.2016.02.001 PMC4850498

[231] Salvatore MF, Apparsundaram S, Gerhardt GA. Decreased plasma membrane expression of striatal dopamine transporter in aging. Neurobiol Aging 2003;24:1147–54. 14643386 10.1016/s0197-4580(03)00129-5

[232] Ishibashi K, Oda K, Ishiwata K, Ishii K. Comparison of dopamine transporter decline in a patient with Parkinson’s disease and normal aging effect. J Neurol Sci 2014;339:207–9. 24462123 10.1016/j.jns.2014.01.015

[233] Troiano AR, Schulzer M, de la Fuente-Fernandez R, Dopamine transporter PET in normal aging: dopamine transporter decline and its possible role in preservation of motor function. Synapse 2010;64:146–51. 19852071 10.1002/syn.20708

[234] Branch SY, Sharma R, Beckstead MJ. Aging decreases L-type calcium channel currents and pacemaker firing fidelity in substantia nigra dopamine neurons. J Neurosci 2014;34:9310–8. 25009264 10.1523/JNEUROSCI.4228-13.2014 PMC4087208

[235] Chan CS, Gertler TS, Surmeier DJ. A molecular basis for the increased vulnerability of substantia nigra dopamine neurons in aging and Parkinson’s disease. Mov Disord 2010;25 Suppl 1:S63–70. 20187241 10.1002/mds.22801

[236] Venkateshappa C, Harish G, Mythri RB, Mahadevan A, Bharath MM, Shankar SK. Increased oxidative damage and decreased antioxidant function in aging human substantia nigra compared to striatum: implications for Parkinson’s disease. Neurochem Res 2012;37:358–69. 21971758 10.1007/s11064-011-0619-7

[237] Reeve A, Simcox E, Turnbull D. Ageing and Parkinson’s disease: why is advancing age the biggest risk factor? Ageing Res Rev 2014;14:19–30. 24503004 10.1016/j.arr.2014.01.004 PMC3989046

[238] Trist BG, Hare DJ, Double KL. Oxidative stress in the aging substantia nigra and the etiology of Parkinson’s disease. Aging Cell 2019;18:e13031. 31432604 10.1111/acel.13031 PMC6826160

[239] Noda S, Sato S, Fukuda T, Tada N, Hattori N. Aging-related motor function and dopaminergic neuronal loss in C57BL/6 mice. Mol Brain 2020;13:46. 32293495 10.1186/s13041-020-00585-6 PMC7092461

[240] McCormack AL, Di Monte DA, Delfani K, Aging of the nigrostriatal system in the squirrel monkey. J Comp Neurol 2004;471:387–95. 15022260 10.1002/cne.20036

[241] Emborg ME, Ma SY, Mufson EJ, Age-related declines in nigral neuronal function correlate with motor impairments in rhesus monkeys. J Comp Neurol 1998;401:253–65. 9822152

[242] Jiang C, Wan X, He Y, Pan T, Jankovic J, Le W. Age-dependent dopaminergic dysfunction in Nurr1 knockout mice. Exp Neurol 2005;191:154–62. 15589522 10.1016/j.expneurol.2004.08.035

[243] Chu Y, Le W, Kompoliti K, Jankovic J, Mufson EJ, Kordower JH. Nurr1 in Parkinson’s disease and related disorders. J Comp Neurol 2006;494:495–514. 16320253 10.1002/cne.20828 PMC2564615

[244] Zetterstrom RH, Solomin L, Jansson L, Hoffer BJ, Olson L, Perlmann T. Dopamine neuron agenesis in Nurr1-deficient mice. Science 1997;276:248–50. 9092472 10.1126/science.276.5310.248

[245] Chu Y, Kompoliti K, Cochran EJ, Mufson EJ, Kordower JH. Age-related decreases in Nurr1 immunoreactivity in the human substantia nigra. J Comp Neurol 2002;450:203–14. 12209851 10.1002/cne.10261

[246] Ma SY, Ciliax BJ, Stebbins G, Dopamine transporter-immunoreactive neurons decrease with age in the human substantia nigra. J Comp Neurol 1999;409:25–37. 10363709 10.1002/(sici)1096-9861(19990621)409:1<25::aid-cne3>3.0.co;2-e

[247] Murty VP, Shah H, Montez D, Foran W, Calabro F, Luna B. Age-related trajectories of functional coupling between the VTA and nucleus accumbens depend on motivational state. J Neurosci 2018;38:7420–7. 30030394 10.1523/JNEUROSCI.3508-17.2018 PMC6104300

[248] Backman L, Lindenberger U, Li SC, Nyberg L. Linking cognitive aging to alterations in dopamine neurotransmitter functioning: recent data and future avenues. Neurosci Biobehav Rev 2010;34:670–7. 20026186 10.1016/j.neubiorev.2009.12.008

[249] Peterson AC, Zhang S, Hu S, Chao HH, Li CR. The effects of age, from young to middle adulthood, and gender on resting state functional connectivity of the dopaminergic midbrain. Front Hum Neurosci 2017;11:52. 28223929 10.3389/fnhum.2017.00052 PMC5293810

[250] Kubis N, Faucheux BA, Ransmayr G, Preservation of midbrain catecholaminergic neurons in very old human subjects. Brain 2000;123 (Pt 2):366–73. 10648443 10.1093/brain/123.2.366

